# Design Strategies for Developing Chiral Macrocyclic Chelators as a Diagnostic Tool

**DOI:** 10.1021/acs.accounts.6c00081

**Published:** 2026-07-16

**Authors:** Yifan Zhu, Ga-Lai Law

**Affiliations:** † Department of Chemistry, 26680 The Hong Kong Polytechnic University, Hung Hom, Hong Kong SAR, China

## Abstract

Macrocyclic chelators have become an indispensable part of the field of molecular imaging due to their ability to form highly stable complexes with various metal ions for diverse diagnostic and therapeutic applications. Among them, DOTA (1,4,7,10-tetra­azacyclo­dodecane-1,4,7,10-tetraacetic acid), which serves as a gold standard in both stability and versatility, is extensively utilized in clinical contexts as contrast agents for a series of imaging modalities, including MRI (magnetic resonance imaging), PET (positron emission tomography), SPECT (single-photon emission computed tomography), and optical imaging. Despite its advantageous properties, in certain cases, DOTA still has limitations to be solved, such as slow radiolabeling kinetics and possible *in vivo* decomplexation, which have led to increased research interest in structural modifications of the DOTA ligand.

To date, the most popular design strategy for DOTA analogues focuses on the modification of pendant arms, which can be simply achieved through the N-derivatization of the tetraaza macrocycle. Another strategy is to modify the macrocyclic ring structure, with the most successful example being the macrocycle pyclen, which contains a pyridine moiety. In 2022, pyclen-based Gd^3+^ complex gadopiclenol was approved by the U.S. FDA (United States Food and Drug Administration) as a next-generation MRI contrast agent for clinical use. However, reported studies on similar structural modifications of macrocyclic rings are relatively rare, as this requires the redesign of synthetic procedures that can be highly complicated.

In this Account, we discuss our design strategies of various macrocyclic chelators with chiral substituents on the macrocyclic ring for different biomedical imaging applications. We start with structural modifications to the frequently used DOTA chelator through the introduction of four symmetrical chiral substituents on its macrocyclic backbone. Results of NMR studies confirmed that the incorporated chiral substituents can prevent the interconversion between two TSAP/SAP regioisomers formed in the complexation process of DOTA with lanthanides. The chiral substituents also provide increased ligand rigidity and can enhance the coordination geometry for better encapsulation of the metal, preventing possible dechelation and transmetalation, thereby achieving higher coordination stability. A series of DOTA analogues with different chiral substituents were studied, and we have revealed that the TSAP/SAP isomeric ratios and pharmacokinetics of chiral DOTA varied with the steric hindrance and polarity of attached chiral groups. In particular, the Gd^3+^ complexes of chiral DOTAs with symmetric aliphatic groups exhibit enhanced relaxivity over that of achiral DOTA, making them ideal contrast agents for MR imaging. Through the subsequent optimization of introduced chiral groups, various improved chelators were then developed for photoluminescence or for MRI.

Next, we focused on further pendant arm modifications of these chiral DOTA platforms. Multiple functionalized pendant arms were attached to the chiral macrocycle as chromophores or targeting vectors, resulting in versatile chiral bifunctional chelators for targeted circularly polarized luminescence (CPL), PET/SPECT, and radiotherapy applications. Moreover, our chiral modifications are not limited to the 12-membered DOTA macrocycle. Recently, we broadened our design strategy to alter the macrocyclic ring sizes, an approach with relatively few literature examples. The chiral analogues of 9-membered NOTA and 10-membered DETA systems were synthesized for ^68^Ga radiolabeling, with the influence of cavity sizes and positions of chiral substitution investigated. Follow-up studies will go deeper into the metal ion selectivity effects of differently sized chiral macrocycles to pave the way for future designs on novel macrocyclic chelators.

## Graphical abstract

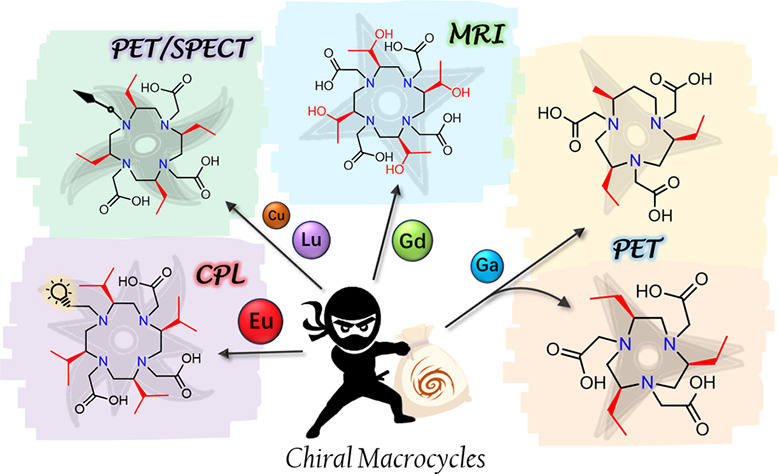

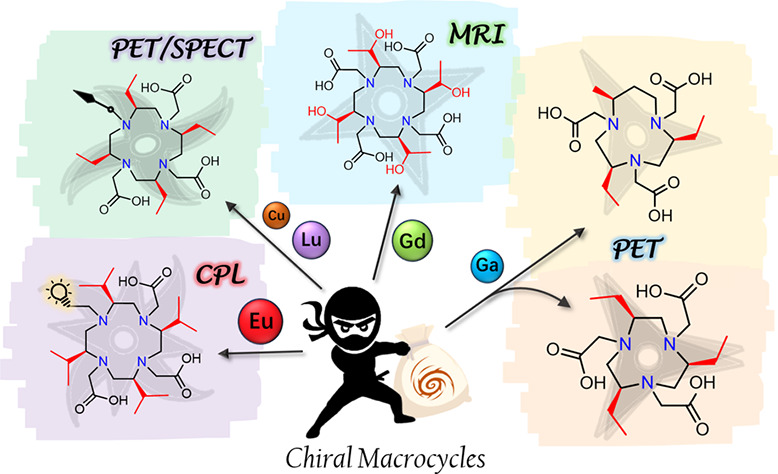

## Key References

-

Dai, L. ; Jones, C. M. ; Chan, W. T. K. ; Pham, T. A. ; Ling, X. ; Gale, E. M. ; Rotile, N. J. ; Tai, W. C.-S. ; Anderson, C. J. ; Caravan, P. ; Law, G.-L. Chiral DOTA chelators as an improved platform for biomedical imaging and therapy applications. Nat. Commun. 2018, 9­(1), 857 10.1038/s41467-018-03315-8 29487362 PMC5829242.^1^ This work reported the synthesis of a series of chiral DOTA chelators with modified macrocyclic backbones. The introduced chiral substituents enhanced the rigidity and stability of these chelators, enabling control of structural isomerism of their lanthanide complexes.
-

Zhang, J. ; Dai, L. ; Webster, A. M. ; Chan, W. T. K. ; Mackenzie, L. E. ; Pal, R. ; Cobb, S. L. ; Law, G. L. Unusual magnetic field responsive circularly polarized luminescence probes with highly emissive chiral europium (III) complexes. Angew. Chem., Int. Ed. 2021, 60­(2), 1004–1010 10.1002/anie.202012133 PMC782114632959961.^2^ In this work, functionalized pendant arms were attached to various chiral DOTA chelators to create targeted CPL luminescence probes. The Eu^3+^ complexes of these modified chiral DOTAs exhibited improved photophysical properties and magnetic CPL responses.
-

Zhang, J. ; Dai, L. ; He, L. ; Bhattarai, A. ; Chan, C.-M. ; Tai, W. C.-S. ; Vardhanabhuti, V. ; Law, G.-L. Design and synthesis of chiral DOTA-based MRI contrast agents with remarkable relaxivities. Commun. Chem. 2023, 6­(1), 251 10.1038/s42004-023-01050-w 37973896 PMC10654417.^3^ Two chiral DOTAs with hydrophilic functional groups were developed as Gd-based contrast agents, showing high water solubility and improved secondary-sphere relaxivity, suitable for potential MRI applications.
-

Zhu, Y. ; Cheng, Z. ; Zhang, J. ; Feng, L. ; Mishra, A. ; de Rosales, R. T. M. ; Brown, R. K. ; Long, N. ; Law, G.-L. Influences of Ring Sizes and Chiral Substituents on Macrocyclic Chelators for Improving ^68^Ga Radiolabeling. JACS Au 2026, 6­(1), 113–123 10.1021/jacsau.5c00946 41614168 PMC12848718.^4^ Chiral analogues of 9-membered NOTA, 10-membered DETA, and 12-membered DOTA chelators were synthesized with detailed radiolabeling, equilibrium, and computational studies to reveal the influence of macrocyclic cavity size and chiral substitution positions on Ga^3+^ coordination properties.

## 1 Introduction

Macrocycles are ubiquitous in nature and are extensively used as chelators to form metal complexes. Classic examples are hemes and chlorophylls featuring porphyrin complex-based structures which display key functional roles such as for oxygen transportation in animals and energy transfer in plants, respectively.^5,6^ Within these natural macrocycles, the central metal ion is firmly encapsulated within the ligand cavity, thereby conserving the coordination chemistry, metal stability, and functions of the biomolecules against harsh physiological environments.^7^ Due to their robustness, this has created a niche for scientists to investigate and mimic the structure and function of these natural macrocyclic systems.^8^ It has been proven that macrocyclic ligands are able to form metal complexes with enhanced thermodynamic stability and kinetic inertness compared to acyclic ligands, a phenomenon commonly known as the macrocyclic effect.^9,10^ In past decades, numerous synthetic macrocyclic ligands, especially macrocyclic polyamines, have been developed for clinical, industrial, and laboratory applications such as molecular imaging, radiotherapy, biosensing, and metal ion separations.^11–18^

The tetraazamacrocycle DOTA (1,4,7,10-tetra­azacyclo­dodecane-1,4,7,10-tetra­acetic acid) and its derivatives stand out as “gold standard” macrocyclic polyamines that form stable lanthanide and transition-metal complexes with high kinetic inertness and unique properties that are well suited for *in vivo* applications including magnetic resonance imaging (MRI), positron emission tomography (PET), single-photon emission computed tomography (SPECT), and optical imaging.^15,19–23^ Several metal complexes of DOTA derivatives have been approved by the U.S. Food and Drug Administration (FDA) as diagnostic probes for clinical use^24^ despite limitations such as slow radiolabeling kinetics and possible decomplexation *in vivo*,^20,25–28^ leading to the design of next-generation ligands with higher inertness, improved coordination properties, or ligand versatility.^29–34^ In this regard, there are three mainstream design strategies for improving macrocyclic polyamines: (1) modifying the pendant arms, (2) modifying the macrocyclic backbone, and (3) altering the macrocyclic ring size (Figure 1).

**Figure 1:**
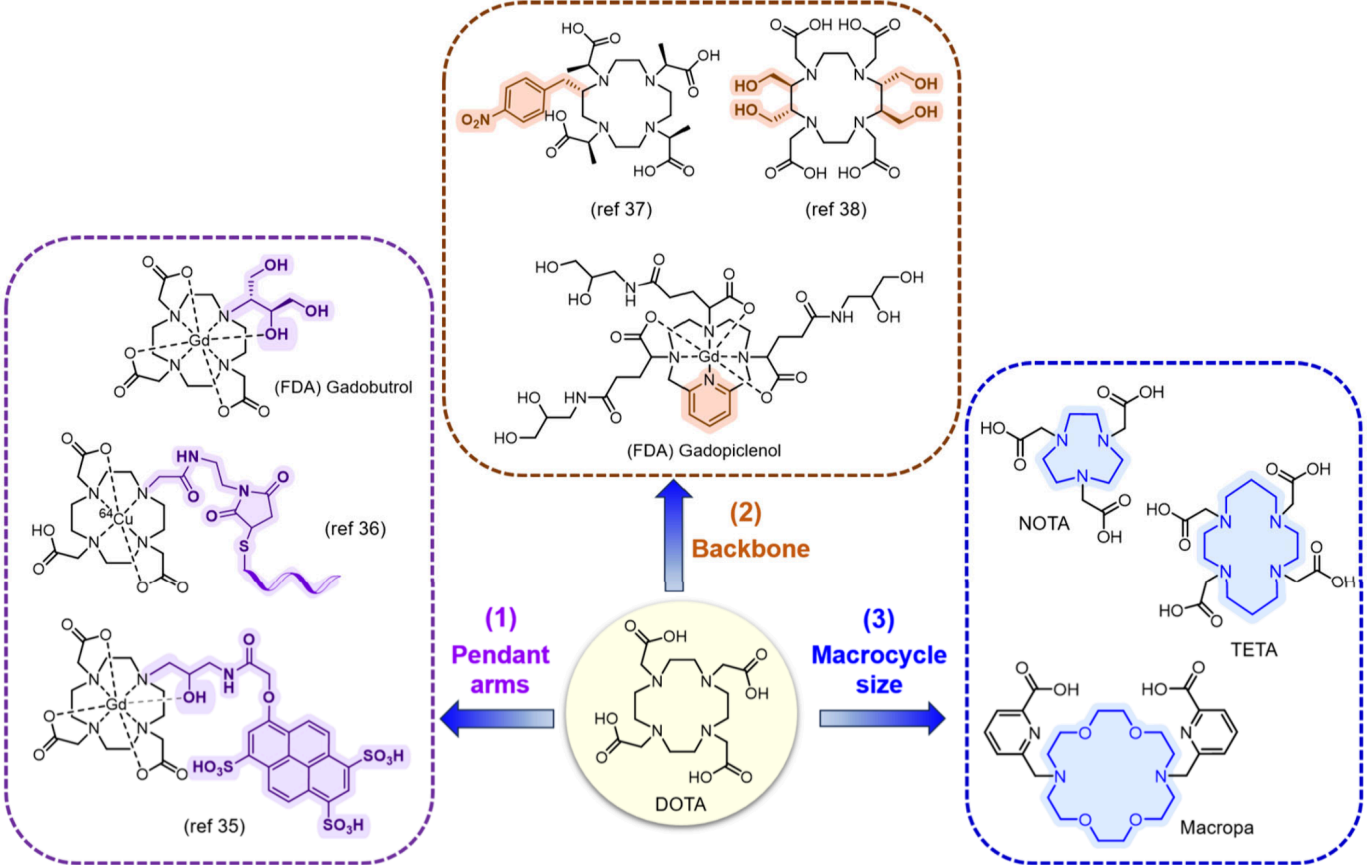
Three strategies for developing improved macrocyclic polyamines for biological applications with examples from the literature^35–38^ or approved by the FDA.

At present, the most common and easiest structural modification for DOTA-based macrocyclic chelators is changing the N-substituent pendant arms. This strategy is prevalent among MRI contrast agents as it improves the water relaxivity of Gd-DOTA complexes by altering the water-exchange mechanics around the Gd^3+^ center,^39,40^ such as FDA-approved gadobutrol and gadoteridol that have hydroxyl-based arms.^40^ Or another example by Gianolio’s group, showed by introducing a trisulfonated pyrene moiety on the DOTA, this provided enhanced relaxivity via increased molecular weight.^35^ Functionalizing pendant arms with a vector could also give rise to bifunctional chelators (BFCs) where the macrocyclic complex is responsible for the imaging and the functional arm imparts specific targeting which is crucial in many imaging modalities. In a typical BFC design, one carboxylic arm is generally covalently conjugated to a cancer-binding antibody, peptide or protein via a linker to enable specific targeting of cancer cells.^36^ In general, the convenience of pendant arm modifications offers extraordinary versatility to the DOTA platform for various imaging applications, and this strategy has been extensively studied.^35,36,41–49^

Modifying the DOTA macrocycle backbone is much more challenging from a synthetic perspective; it also changes the ligand’s preorganization to afford distinct coordination geometry. In earlier reports, bulky alkyne groups or chiral substituents were incorporated on the macrocyclic ring to create new MRI contrast agents with improved relaxivity.^37,50^ More recently, a chiral DOTA analogue with two sets of hydroxyl-methylene substituents on the adjacent macrocyclic carbons was also reported, exhibiting high structural rigidity and hydrophilicity.^38^ The most significant improvement is FDA-approved MRI contrast agent gadopiclenol: the pyridine moiety on the macrocycle backbone gives it significant stability and a great increase in the water relaxivity of its Gd^3+^ complexes.^51,52^ However, similar structural engineering of macrocyclic ring is still limited by synthetic challenges, with fewer literature examples than for the pendant arm modifications.

Hancock et al. reported in the 1980s that the cavity size and coordination geometry of macrocyclic ligands play a crucial role in the formation kinetics and thermodynamic stability of their complexes.^53^ This triggered researchers to investigate macrocyclic chelators with different ring sizes to seek better metal ion selectivity. A representative example is the 9-membered chelator NOTA (1,4,7-triaza­cyclononane-1,4,7-triacetic acid). With a smaller cavity size than DOTA, NOTA exhibits ideal binding affinity to smaller transition and post-transition metal ions like Ga^3+^ for ^68^Ga-labeled PET imaging.^54–62^ A larger macrocyclic ring, the 14-membered TETA (1,4,8,11-tetra­azacyclotetra­decane-1,4,8,11-tetraacetic acid), has also demonstrated excellent compatibility with Cu^2+^ ions due to its favorable coordination geometry.^63–65^ Other examples, like the 18-membered macropa, have emerged as novel chelators for large trivalent radiometals like ^225^Ac, ^133^La, ^177^Lu, ^213^Bi, ^212^Pb, ^131^Ba, and ^223/224^Ra.^66–72^ However, it is worth noting that, to date, DOTA is the only macrocyclic ring adopted in the FDA-approved clinical contrast agents. The research on macrocyclic chelators of different sizes is still a diamond in the rough that requires further development to fulfill their full potential for biological applications.

When designing DOTA-based macrocyclic chelators for lanthanide coordination, an important phenomenon that should be taken into consideration is the stereoisomerism of the resulting complexes. It is well documented that there are four conformational isomers present in aqueous solution: two pairs of enantiomers with square antiprismatic (SAP, corner/side) and twisted square antiprismatic (TSAP, corner/side) geometries (Figure 2). The SAP and TSAP conformations arise through the rotation of carboxylate pendant arms,^73,74^ whereas the side and corner conformations are categorized by the geometry of the tetraaza macrocycle.^29^ In nonchiral Ln-DOTA systems, the four conformational isomers are interconvertible on a time scale of 10–100 ms through rotation of the acetate arms or inversion of the macrocyclic ring.^75–77^ With different steric crowding and activation energy for conformation exchange, SAP and TSAP isomers exhibit water-exchange rates that can differ by up to 2 orders of magnitude, resulting in distinct MRI contrast performances.^19,73,78–80^ They can also generate multiple resonances in paramagnetic NMR spectroscopy, hindering the accurate determination of protein structures.^38,81^ Therefore, researchers are devoted to controlling the ratio of conformational isomers in Ln-DOTA complexes.^19,82^ Similar structural isomerism was also found in various metal complexes of the other macrocyclic ligands, such as the NOTA systems.^83–85^

**Figure 2:**
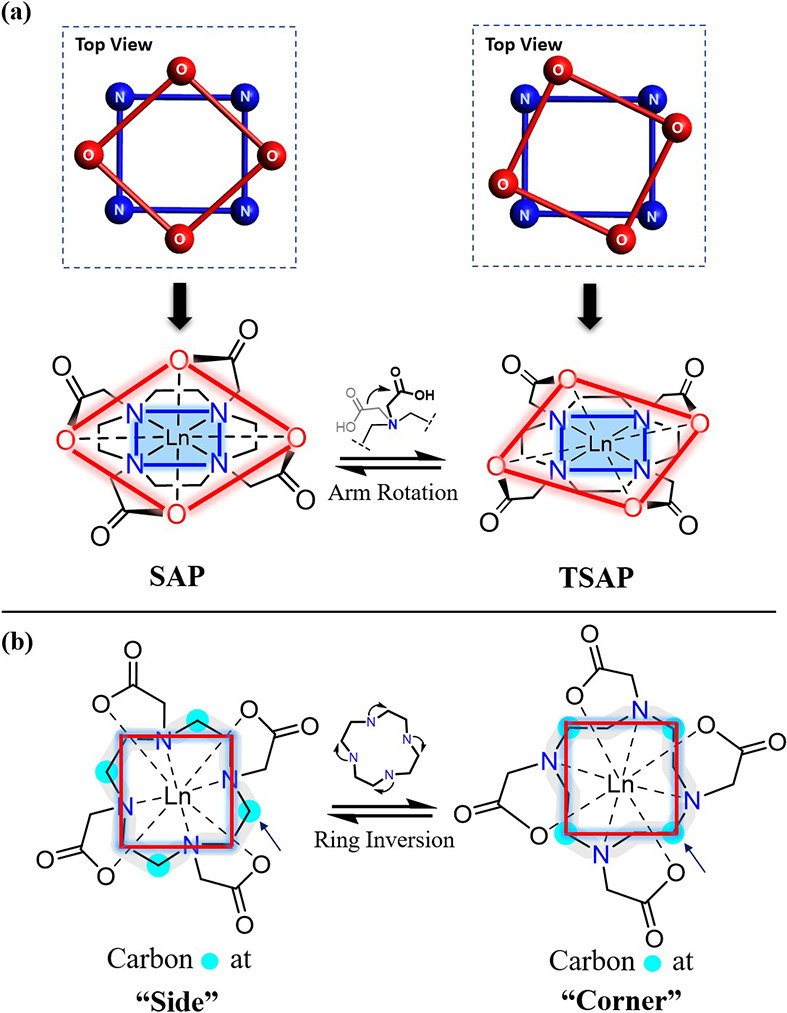
Illustration of the geometric isomerism in Ln-DOTA complexes. (a) SAP and TSAP isomerism and (b) “side” and “corner” isomerism.

The selective formation of SAP and TSAP isomers can be achieved by enhanced ligand rigidification of DOTA analogues.^73,82,86–88^ When introducing bulky or chiral substituents on the pendant arms or the macrocyclic ring of DOTA chelator, the resulting Ln^3+^ complexes can be locked into either SAP or TSAP geometry. However, compared to the widely studied chiral modifications on acetate pendant arms, literature examples of chiral substitution on the DOTA macrocycle are relatively limited,^38^ likely due to synthetic challenges.^86,89,90^ In our original design of chiral DOTA systems, we employed an optimized synthetic route with simplified steps, convenient purification, and high overall yields.^1^ It enabled the gram-scale preparation of various rigid chiral DOTA chelators bearing symmetric macrocyclic backbones for in-depth studies of their coordination chemistry with different metal ions. We then explored the other modification strategies, including pendant arm modifications and variations in macrocyclic ring sizes.^2–4^ These efforts led to the development of a series of chiral macrocyclic chelators for biological applications, such as sensors and imaging probes.

## 2 Modification of Macrocyclic Backbones.

In our first work to improve DOTA, we focused on its macrocyclic ring framework for an enhanced ligand preorganization (Figure 3). With our optimized synthetic route featuring a tetrameric cyclization reaction, chiral DOTA ligands **L1**–**L5** were synthesized with methyl, ethyl, isobutyl, benzyl, and aminobutyl groups, respectively^1^ (Figure 4). The chiral substituents with varied steric hindrance gave rise to different properties such as polarity, ligand rigidity, and hence different influences on the coordination isomers of its metal complexes. This led to further designs of ligands **L6**–**L8** to explore controlling the coordination isomers.

**Figure 3:**
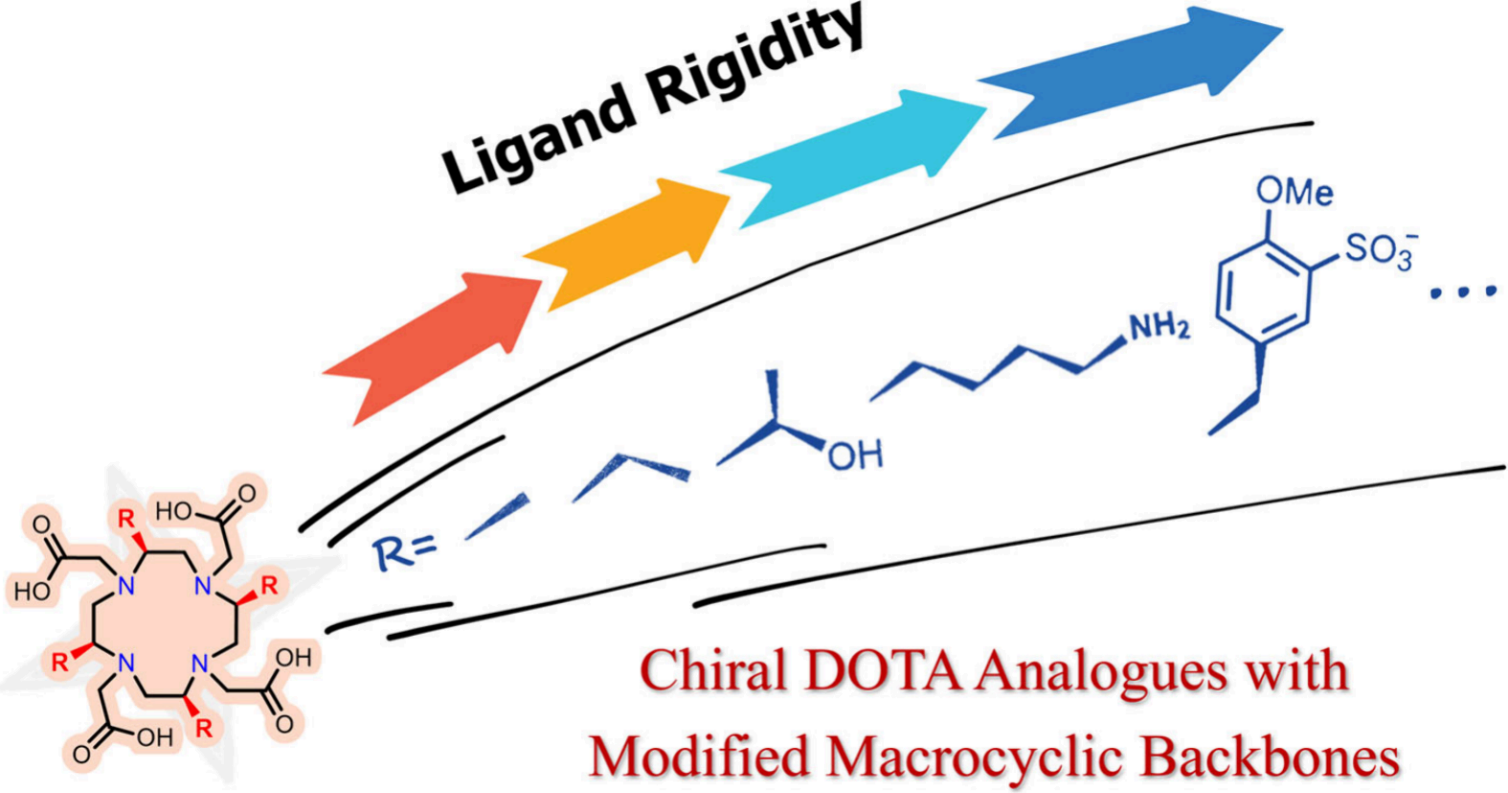
Design of chiral DOTA analogues with modified macrocyclic backbones in order of increased steric hindrance.^1,3,91^

**Figure 4:**
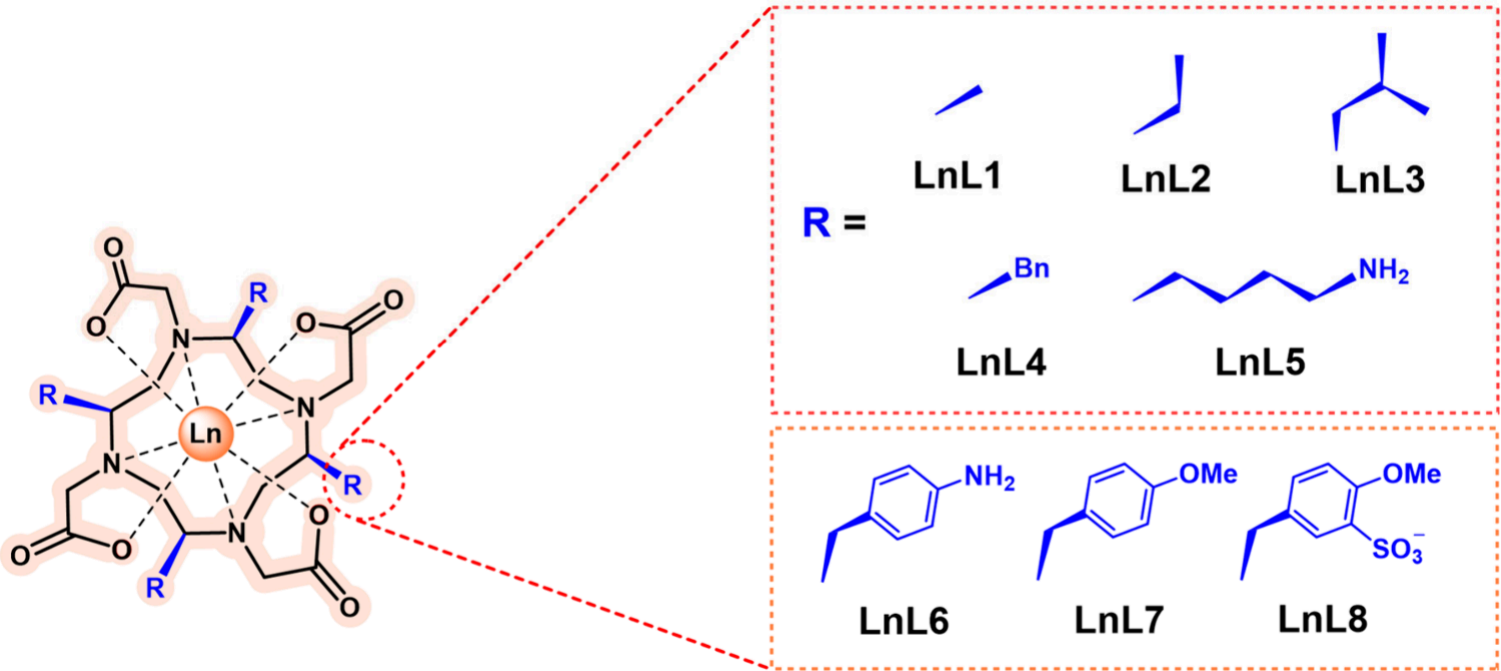
Structures of the lanthanide (Ln) complexes with chiral DOTA ligands **L1**–**L8** bearing diverse chiral substituents.^1,91^

According to the crystal structure of one of the resulting chiral macrocyclic complexes, **GdL4**, while the carboxylic pendant arms were pulled toward the metal center for coordination, all four chiral substituents on the macrocycle pointed in the opposite direction, creating a more rigid framework and simultaneously forming a shield to protect the metal surface.^1^

We then studied the structural isomerism of our chiral DOTA complexes with Eu^3+^ and Yb^3+^, which showed that the extra steric hindrance from the chiral substituents restricts ring inversion, thereby preventing the interconversion between the corner and side pairs (Figure 5). As a result, the conformation of TSAP and SAP isomers was “locked” after complexation, enabling chromatographic isolation. According to our ^1^H NMR studies, the isomeric ratio of our chiral DOTA complexes correlated strongly with the steric hindrance of chiral substituents (Figure 6): the TSAP isomers normally prefer sterically crowded environments at the metal center.^92^ The only exception is **EuL5**, where only the SAP isomers were observed, which might be attributed to the change in the solvation environment around the polar amine substituent.

**Figure 5:**
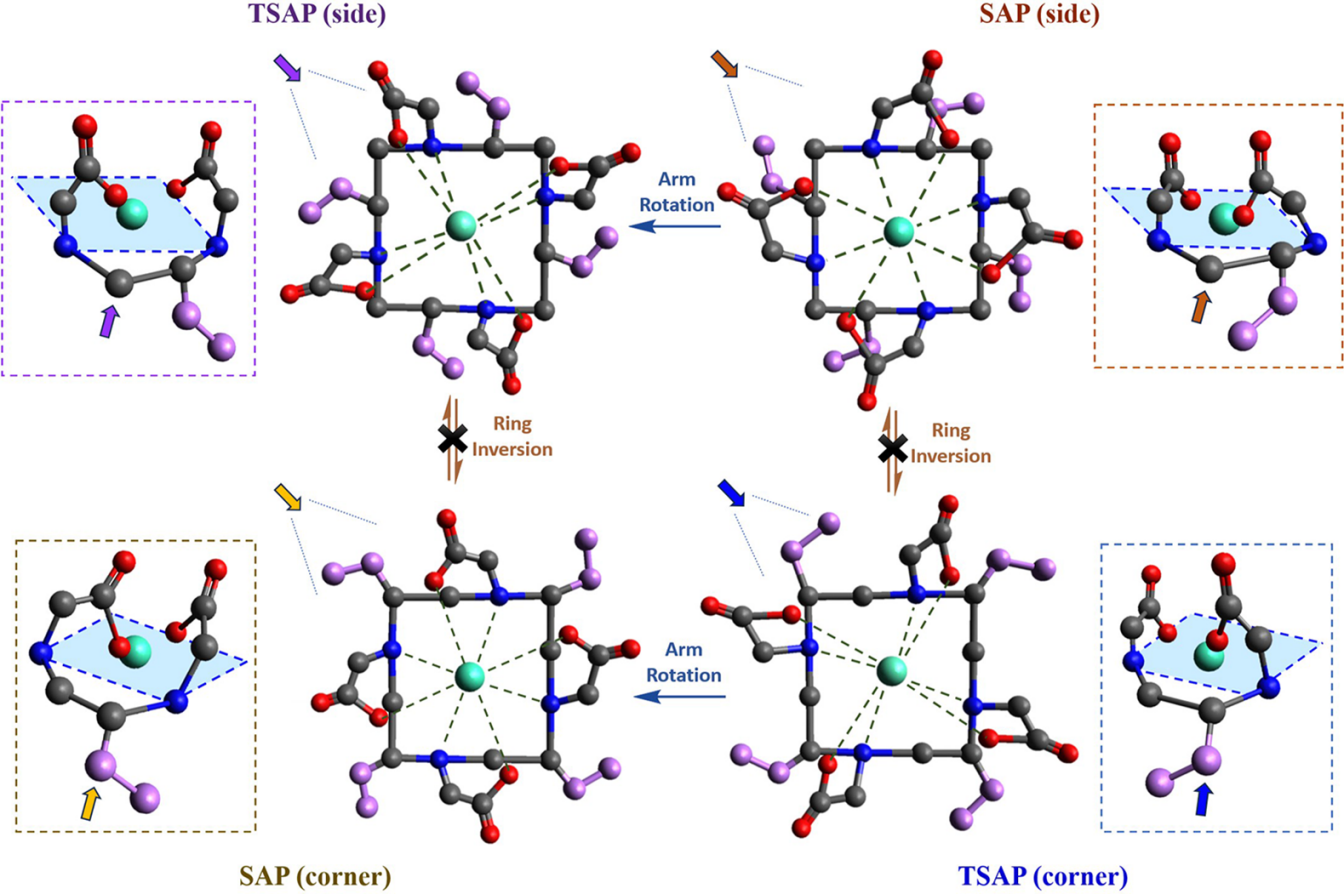
Restricted isomerism of our chiral DOTA complexes with an enlarged corner view of each macrocyclic ring in the dashed boxes. (Planes formed by the four nitrogen atoms are highlighted in blue; some atoms are omitted for clarity.)

**Figure 6:**
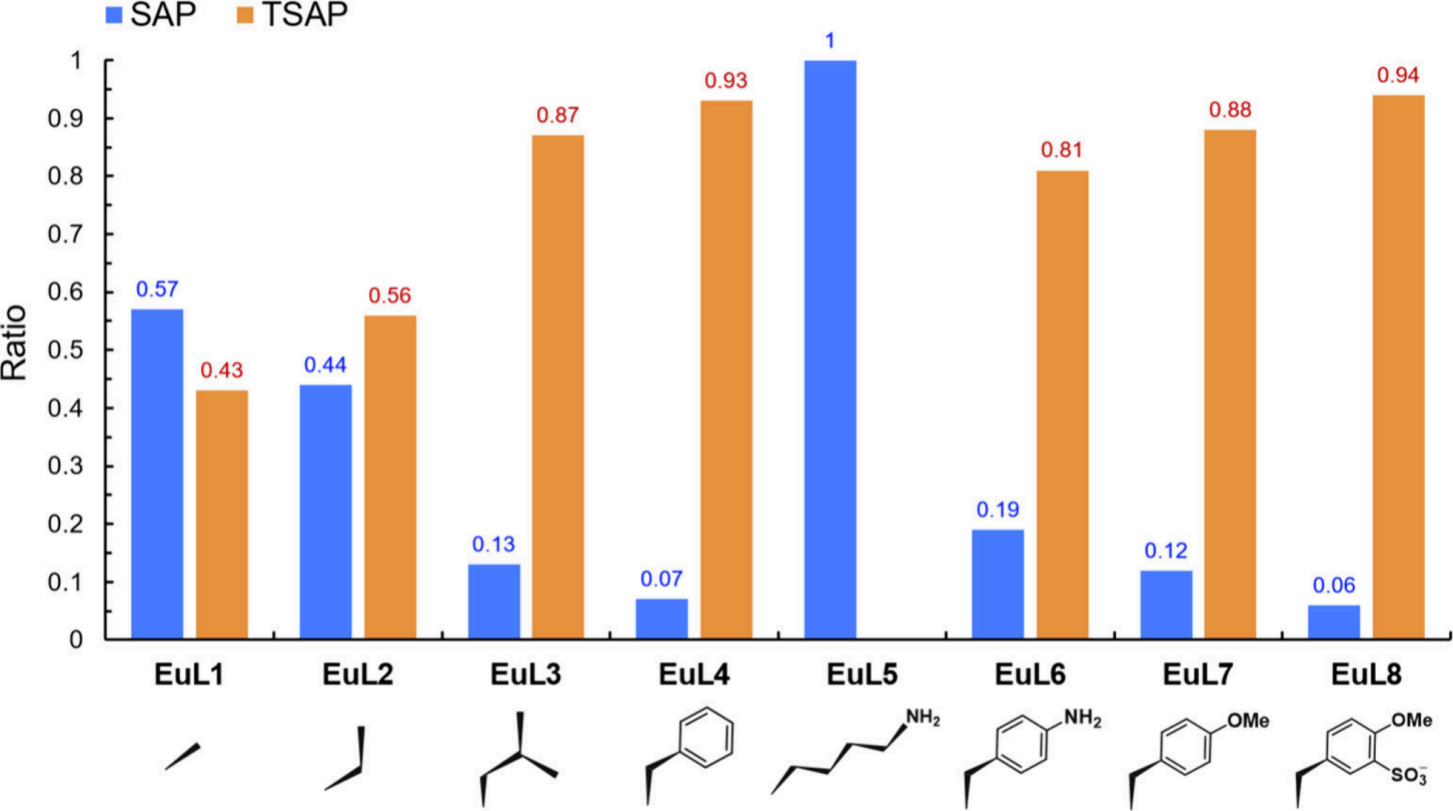
SAP/TSAP isomer ratios in the Eu^3+^ complexes of chiral DOTA **L1**–**L8** according to the integration of ^1^H NMR signals.^1,91^

When MRI contrast agents are evaluated, one of the most important parameters is relaxivity, which can be influenced by the coordination isomers formed in Gd^3+^ complexation. For DOTA-based MRI contrast agents, the TSAP isomers exhibit faster water exchange kinetics, hence accounting for more relaxivity enhancement than the SAP isomers as they fall in the optimal window for water exchange.^79,93^ Our chiral **GdL1**–**GdL5** showed very fast water exchange rates with a much shorter water residence time (τ_M_^310 K^) than that of achiral Gd-DOTA. The *r*_1_ and *r*_2_ values of achiral Gd-DOTA, **GdL1**, **GdL2**, and **GdL5** were measured at 1.4 T, 37 °C in water, 4.5% (w/v) human serum albumin (HSA) solution, and human plasma (Table 1). Notably, we synthesized and separated four isomers of **GdL2**, namely, **GdL2A** (SAP), **GdL2B** (TSAP), **Gd­(** ***R*** **)­L2A** (SAP), and **Gd­(** ***R*** **)­L2B** (TSAP), to investigate their influences. Our chiral DOTA complexes demonstrate moderately higher *r*_1_ relaxivities than Gd-DOTA, showing potential to bring about improved imaging performance in a clinical setting.

**Table 1:** Relaxivities of Gd^3+^ Complexes in Water, HSA, and Human Plasma Measured at 1.4 T, 37 °C^1^

	Water		4.5% w/v HSA		Human Plasma	
Sample	*r*_1_	*r*_2_	*r*_1_	*r*_2_	*r*_1_	*r*_2_
**GdL1**	4.1	5.1	4.5	5.3	4.6	5.5
**GdL2A**	4.2	5.5	4.7	5.5	4.7	5.6
**GdL2B**	3.9	4.9	4.8	6.0	4.9	6.1
**Gd(** ***R*** **)L2A**	3.9	5.0	4.5	4.9	4.6	4.8
**Gd(** ***R*** **)L2B**	3.9	4.9	4.8	6.1	4.9	6.1
**GdL5**	4.1	5.9	5.1	6.3	5.1	6.3
**GdL8** ^a^	6.8	/	/	/	/	/
Gd-DOTA	3.2	3.2	4.1	4.8	4.0	4.0

a Measured in water at 1.5 T, 37 °C from ref 91.

The kinetic inertness of GBCAs is an important parameter that can imply *in vivo* stability. The stabilities of the SAP and TSAP isomers of **GdL2** were evaluated separately. Gd biodistribution studies in BALB/c mice revealed that, different from the rapid and exclusive elimination of normal Gd-DOTA *via* the renal pathway as previously reported, the biodistribution profiles of our chiral DOTA complexes varied with the bulkiness of their chiral macrocyclic backbones (Figure 7).

**Figure 7:**
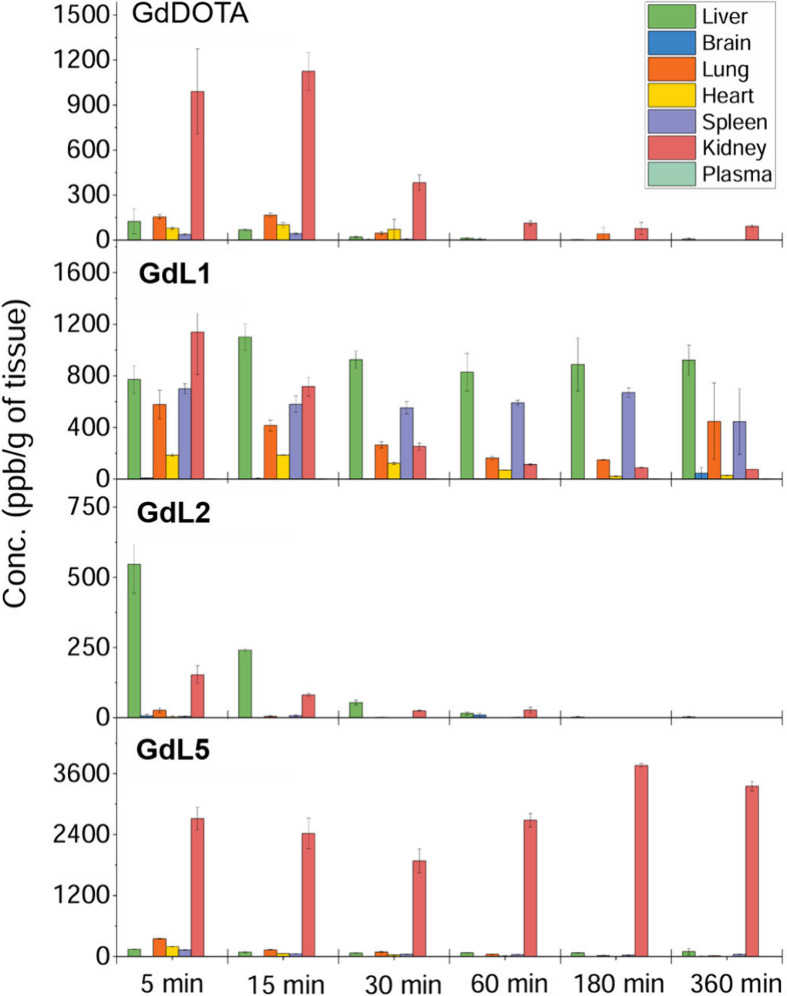
Biodistribution profile of different Gd^3+^ complexes in mice over time. Reproduced from ref 1. Available under a CC BY 4.0 license. Copyright 2018 The Authors.

Although **L3** and **L4**, with bulky chiral substituents, result in a high abundance of TSAP isomers that are beneficial as GBCAs, they are not water-soluble. Therefore, we modified the four chiral benzyl groups on ligand **L4** to give **L6**–**L8** with improved water solubility.^91^ **GdL8**, with four hydrophilic sulfonyl groups, demonstrated the highest TSAP/SAP ratios and *r*_1_ relaxivity, making it ideal for GBCA applications (Table 1).

Moreover, the aromatic chiral substituents on **L7** and **L8** which yielded one dominant isomer (TSAP) in their lanthanide complexes make them suitable for circularly polarized luminescence (CPL) applications.^94^ CPL is a photophysical phenomenon of chiral molecular systems characterized by a high luminescence dissymmetry factor (g_lum_) which reflects the difference in emission intensities between right and left circularly polarized light.^95^ It is useful for probing chiral information in biological systems.^96,97^ Chiral organic compounds normally have g_lum_ values ranging from 10^–4^ to 10^–3^, while our chiral lanthanide complexes can yield g_lum_ > 10^–1^ as they enable CPL emission from the magnetic-dipole-allowed *f*–*f* transitions.^94,98^ The TSAP isomer of **TbL7** showed a high g_lum_ value of 0.285 at 542.5 nm (Figure 8a) from its magnetic-dipole-allowed transition (^5^D_4_ → ^7^F_5_, Δ*J* = 1). Similarly, **TbL8** also displays comparable CPL properties, with a g_lum_ value of 0.241 at 542.5 nm (Figure 8b). The measured g_lum_ values for both compounds are among the highest reported for Tb^3+^ complexes.^99^

**Figure 8:**
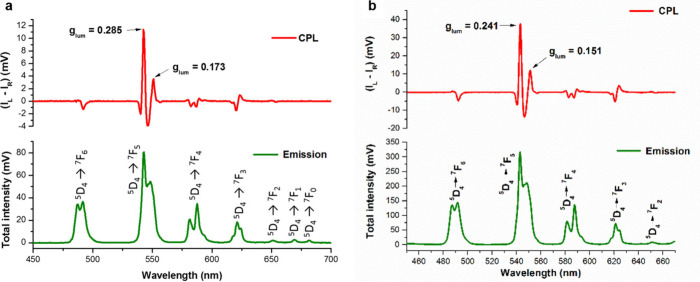
Total emission (green) and CPL (red) spectra for the TSAP isomers of (a) **TbL7** and (b) **TbL8**. Reprinted with permission from ref 91. Copyright 2019 American Chemical Society.

In our follow up studies, hydrophilic chiral groups were selected as functional substituents to enhance the hydrophilicity as MRI contrast agents.^3^ The overall relaxivity of GBCAs is contributed by inner-sphere, secondary-sphere, and outer-sphere relaxivities.^30^ In practice, the mainstream approach is to enhance the inner-sphere relaxivity by increasing the rotational correlation time τ_R_.^100^ However, the contribution from second-sphere relaxivity is generally considered minor unless there are hydrophilic phosphate or hydroxyl groups around the Gd^3+^ ion.^101^ Our **L9** was optimized through conjugating **L5** with aromatic sulfonic acid substitution (Figure 9). Besides improving water solubility, the aromatic substituents also bring about higher molecular weight and accelerated secondary-sphere water exchange.^102^ **L10** features four hydroxy-functionalized chiral groups around the macrocycle for enhancing both inner-sphere and second-sphere relaxivities. All of the measured compounds have much higher relaxivity than Gd-DOTA in both water and 4.5% HSA solution (Table 2).

**Figure 9:**
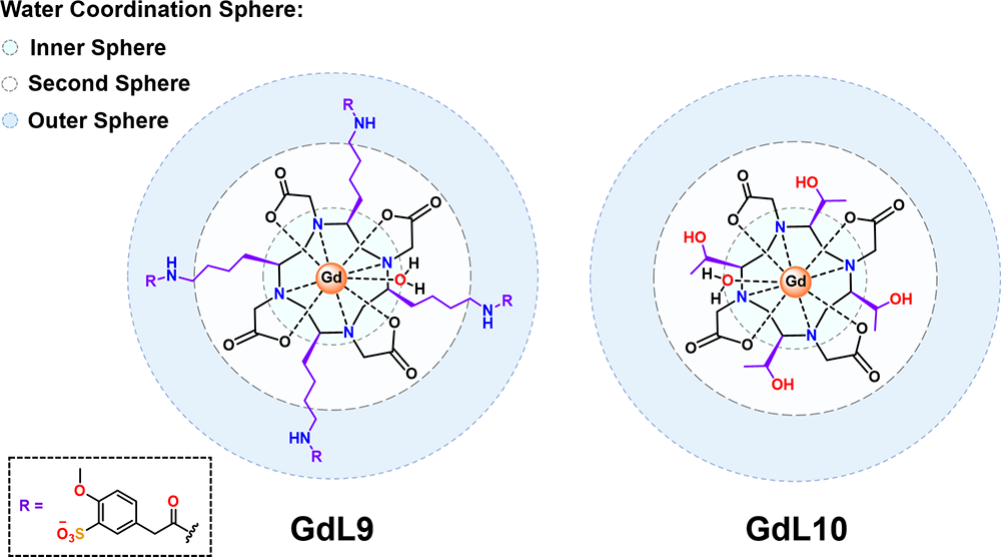
Gd^3+^ complexes of ligand **L9** and **L10** with backbone modifications for enhanced water solubility and relaxivity with their water coordination spheres.^3^

**Table 2:** Relaxivities of the Gd^3+^ Complexes of **L9** and **L10** under 1.4 T, 37 °C, pH 7 ^3^

Complex	*r*_1_ in H_2_O	*r*_2_ in H_2_O	*r*_1_ in 4.5% HSA	*r*_2_ in 4.5% HSA
**(TSAP) GdL9**	7.4 (0.1)	8.4 (0.1)	12.4 (0.8)	16.3 (1.5)
**(SAP) GdL9**	14.5 (0.3)	20.0 (0.2)	17.5 (1.0)	51.9 (2.8)
**GdL10**	5.2 (0.2)	6.1 (0.2)	5.7 (0.4)	7.5 (0.6)
**Gd-DOTA** ^a^	3.2	3.2	4.1	4.8

a Data recorded in ref 1. The standard deviation is shown in parentheses.

Subsequently, pH-dependent *r*_1_ relaxivity measurements of **GdL9** and **GdL10** were conducted. The *r*_1_ relaxivity profiles obtained over pH 2 to 11 had no significant changes, suggesting that no catalyzed proton exchange took place. This result indicates that the considerable improvement of relaxivity for **GdL9** and **GdL10** with hydrophilic functional chiral groups resulted from the enhanced contribution of the secondary and outer-sphere relaxivity. In further kinetic and biodistribution studies, **GdL9** and **GdL10** also demonstrated better kinetic inertness and longer retention in plasma compared with Gd-DOTA.

## 3 Pendant Arm Modifications

Next, we revisited pendant arm functionalization on our chiral DOTA platforms. Many functionalized pendant arms have been developed to improve the properties of DOTA analogues, including chromophores in luminescent applications,^104–106^ linkers for vector conjugation in targeted radiopharmaceuticals,^107–109^ and molecular switches as responsive probes for sensor and imaging applications^110–112^ (Figure 10). Our designs of functionalized chiral DOTA focus on two directions: (1) incorporating a sensitizing unit into our pendant arm to develop luminescent probes or (2) adding functional groups to form a conjugation handle to create BFCs with conjugated targeting vectors.

**Figure 10:**
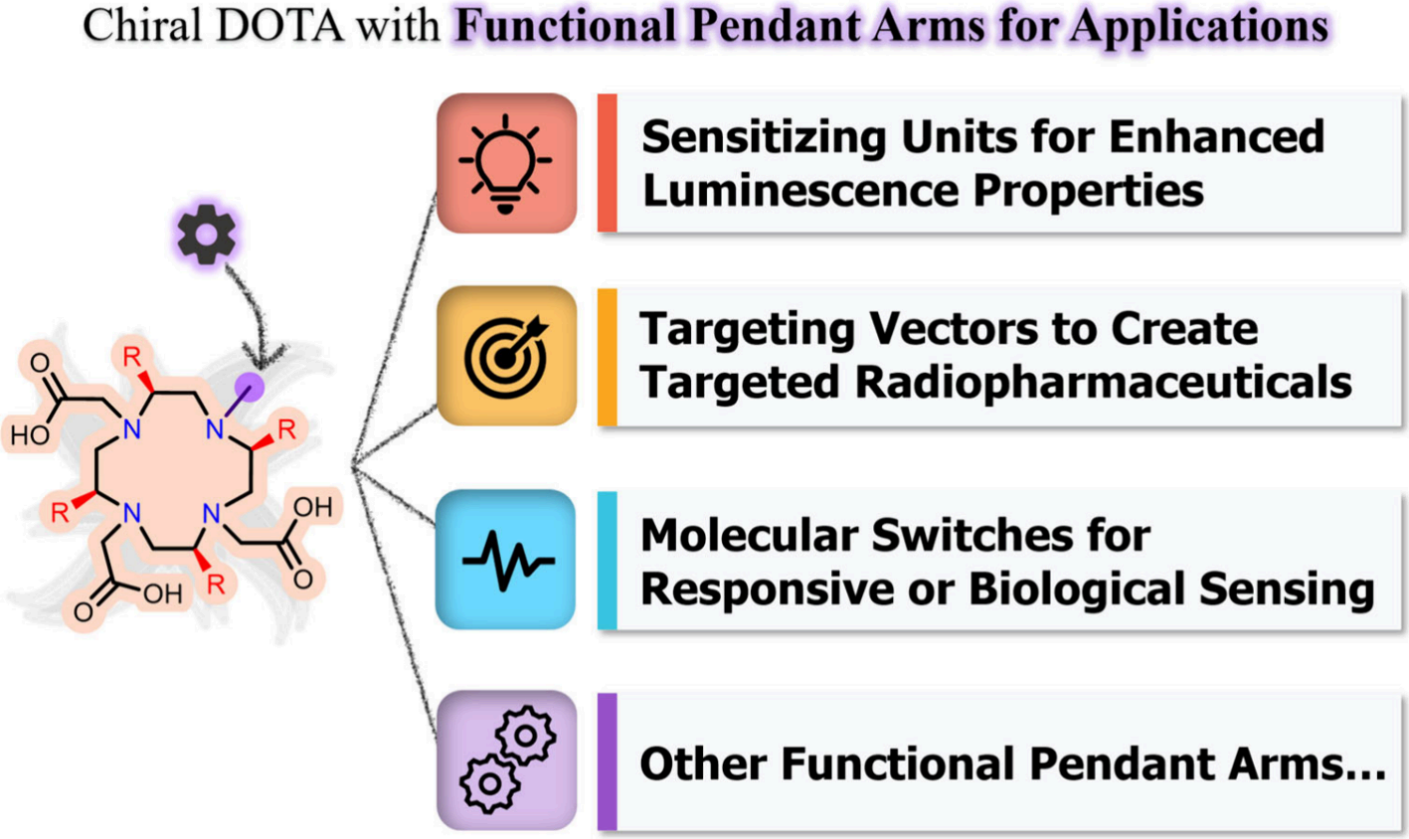
Design of chiral DOTA with functional pendant arms for different applications.^2,103^

On the basis of this strategy, we introduced a chelating chromophore on the modified pendant arm to enhance the luminescence and also water solubility of our complexes to improve upon the properties observed for **TbL7** and **TbL8** complexes which were poorly emissive although displayed CPL properties. Various water-soluble aliphatic chiral DOTA chelators, namely **L12**–**L16** (Figure 11a), were synthesized for luminescence studies;^2^ the extra carboxylic linker also provides a site for bioconjugation. As a proof of concept for developing target-specific bioprobes,^113^ we further designed ligand **L17** that is conjugated to a mitochondria-specific, cell-penetrating peptoid (CPPo), *N*pmb-*N*Lys-*N*pcb-*N*Lys-NH_2_. Due to the ease of synthetic incorporation of bioconjugation handles and initiated by our earlier studies, which revealed our chiral DOTAs to exhibit improved radiolabeling efficacy with ^64^Cu and ^177^Lu^1^, we further developed BFC designs for targeting vectors that are necessary for radiopharmaceutical applications. This led to our designs of **L18** and **L19** (Figure 11b),^103^ where **L18** features a cyclic Arg-Gly-Asp (RGD) peptide conjugated on the pendant arm for binding integrin receptors on cell surfaces,^114–116^ while **L19** has a pendant arm containing a tosyl group which interacts with bovine serum albumin protein^117^ and also serves as a bioconjugation site.

**Figure 11:**
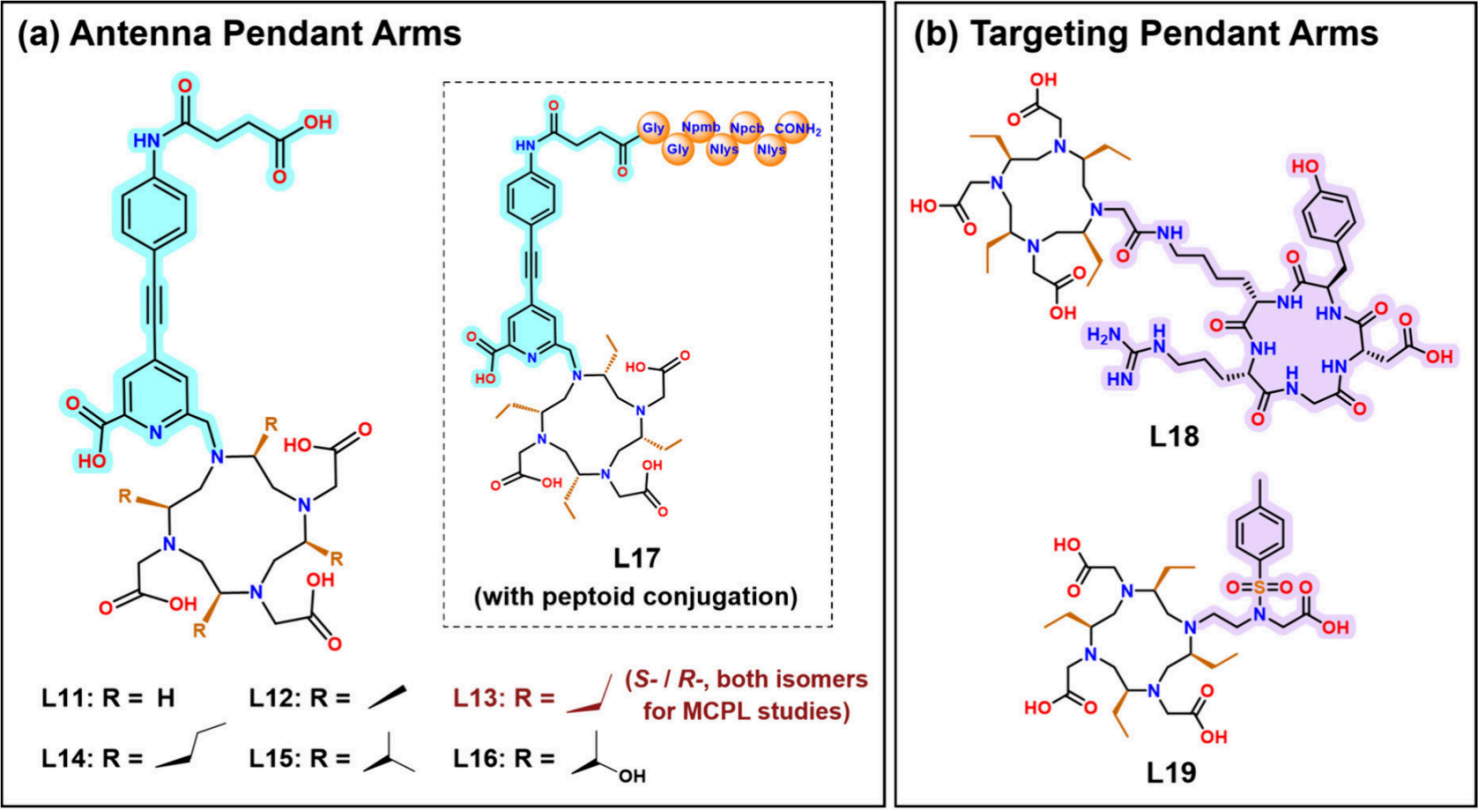
Structures of (a) ligands **L11**–**L17** with functional sensitizing units for luminescent probes^2^ and (b) ligands **L18** and **L19** with targeting vectors as BFCs for PET/SPECT applications.^103^

The luminescence properties of Eu^3+^ complexes of **L12**–**L17** were studied (Table 3). All of the chiral compounds listed in Table 3 are in the SAP form with **(TSAP)­EuL13** being the only exception, which is selected to investigate the influence of coordination geometries. **(SAP)­EuL11–EuL17** with varied chiral backbones did not exhibit significantly different quantum yields, while **(TSAP)­EuL13** has a much lower quantum yield. This difference can be explained by the higher sensitization efficiency (η_sens_) of the SAP isomers (80–90%). In general, the luminescent quantum yields of the chiral **EuL12–EuL17** are much higher than that of nonchiral **EuL11**. This is attributed to the lack of luminescence quenching from coordinating water molecules in the first coordination sphere, as calculated from Parker’s and Horrocks’ equations^118,119^ and also due to the chiral groups further shielding the Eu^3+^ metal center. **EuL12–EuL17** not only have high quantum yields but also comparable g_lum_ values. The highest g_lum_ observed was 0.240 of **EuL15**, belonging to its ^5^D_0_ → ^7^F_1_ transition.

**Table 3:** Photophysical Properties of **EuL11–EuL17** Measured in 0.1 M HEPES, pH 7.3, at 298 K, Excited at 350 nm, with a 380 nm Long-Pass Filter^2^ ^a^

	EuL11	EuL12	(SAP) EuL13	(TSAP) vEuL13	(*R*)EuL13	EuL14	EuL15	EuL16	(*R*)EuL17
**Φ(%)** ^a^	18.4 ± 0.4	27.0 ± 0.3	28.0 ± 1.5	19.8 ± 1.8	26.3 ± 0.6	25.6 ± 0.8	24.1 ± 0.7	26.7 ± 0.2	31.3 ± 1.1
**τ**_**H2O**_ **(ms)** ^b^	0.976 ± 0.09	1.09 ± 0.08	1.30 ± 0.02	1.06 ± 0.02	1.21 ± 0.05	1.35 ± 0.006	1.33 ± 0.006	1.26 ± 0.02	1.39 ± 0.004
**τ**_**D2O**_ **(ms)** ^b^	1.71 ± 0.08	1.93 ± 0.007	2.14 ± 0.01	1.65 ± 0.09	2.14 ± 0.004	2.21 ± 0.005	2.15 ± 0.002	2.10 ± 0.003	2.06 ± 0.003
**Q** ^c^	0.23	0.18	0.06	0.10	0.13	0.05	0.04	0.08	–0.02
**Q** ^d^	0.14	0.10	–0.01	0.03	0.05	–0.02	–0.03	0.01	–0.08
**Φ** ^**Eu**^_**Eu**_ **(%)**	24.3 ± 1.8	29.2 ± 1.4	30.9 ± 1.5	26.6 ± 1.9	32.2 ± 0.7	32.5 ± 0.7	32.4 ± 0.6	31.9 ± 0.2	33.2 ± 0.2
**η**_**sens**_ **(%)**	74.6 ± 3.4	92.4 ± 4.1	90.3 ± 1.9	74.4 ± 3.1	81.6 ± 3.4	78.8 ± 1.3	74.2 ± 3.4	83.7 ± 1.5	94.5 ± 2.5
**R** ^e^	2.61	2.53	2.54	2.50	2.48	2.54	2.52	2.45	2.55
**B** ^f^	3312	4860	5040	3564	4734	4608	4338	4806	5634

a **EuL11–EuL17** are all in the SAP form, except **(TSAP)­EuL13**.

b Relative to quinine sulfate in 0.1 M H_2_SO_4_ (λ_ex_ = 350 nm, Φ = 0.577). Estimated errors in the quantum yield and lifetime are ±15% and ±10%, respectively.

c Measuring the ^5^D_0_ → ^7^F_2_ transition.

d Calculated with Parker’s equation.^118^

e Calculated with Horrocks’ equation.^119^

f I­(^5^D_0_ → ^7^F_2_)/I­(^5^D_0_ → ^7^F_1_).^120^

g B = ε_350 nm_Φ.^121^

The magnetic circularly polarized luminescence (MCPL) responses of **EuL12–EuL17** were also examined under static external magnetic fields. The results showed an obvious linear increase in g_lum_ values from the magnetic dipole-allowed ^5^D_0_ → ^7^F_1_ transition with increasing magnetic field strength (Figure 12). In particular, for the **(** ***S*** **)**- and **(** ***R*** **)-SAP-EuL13**, a 20% enhancement in g_lum_ values was obtained when the magnetic field increased from 0 to 1.4 T at room temperature. It is also worth noting that a linear trend between Δg_lum_ and the applied magnetic field strengths was observed. The high sensitivity and enhancement to the magnetic field enable these complexes to be suitable as magneto-optical probes.^122,123^

**Figure 12:**
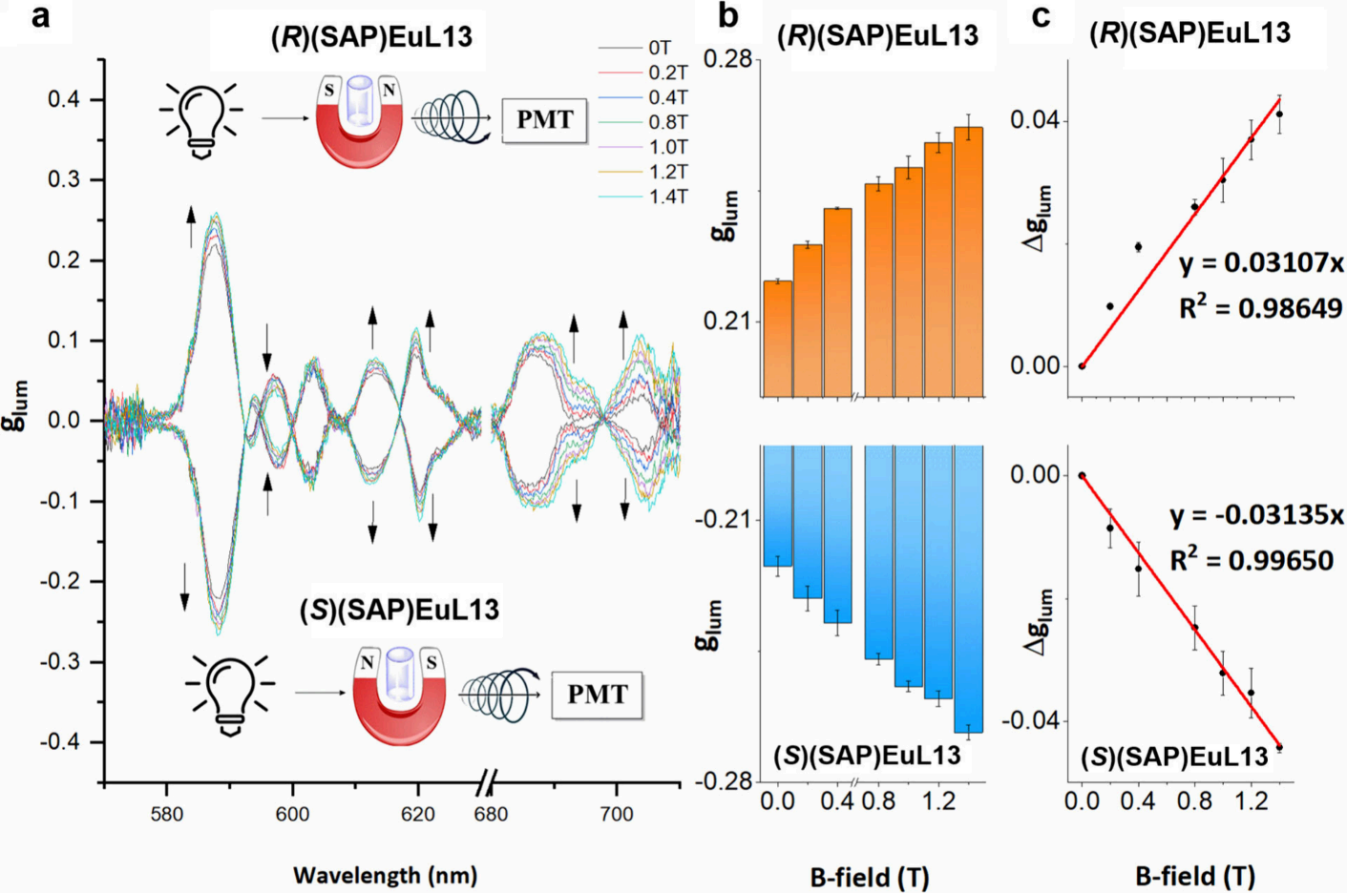
(a) Magnetic-field-dependent g_lum_ spectra of **(*S*)-** and **(*R*)-SAP-EuL13** in 0.1 M HEPES buffer, pH 7.3, excited at 350 nm at room temperature. Arrows indicate the trends in g_lum_ changes; inserted diagrams show the direction of the applied magnetic field. (b) g_lum_ and (c) Δg_lum_ as functions of magnetic field strength for the ^5^D_0_ → ^7^F_1_ transition. Reproduced from ref 2. Available under a CC BY 4.0 license. Copyright 2020 The Auhtors.

The potential of **L18** and **L19** as radiopharmaceutical chelators was evaluated through cold and hot radiolabeling studies with frequently used metal ions in diagnostic and therapeutic radiopharmaceuticals, along with the chiral ethyl ligand **L13** that can also be considered as BFCs due to its extra bioconjugation site. Conducted for our chiral BFCs**.** **L18** was selected for ^64^Cu^2+^ radiolabeling because it showed outstanding Cu^2+^ complexation kinetics. An extremely high radiolabeling yield of >99% was obtained at pH 6.05 after heating at 40 °C for 30 min. Nonadentate ligand **L13** offers a saturated coordination sphere for lanthanide ions with improved stability, thus it was used to examine the radiolabeling efficacy with ^177^Lu^3+^. As expected, effective ^177^Lu labeling was achieved with a yield of 96% after incubating at 45 °C for 15 min (pH 6.0). This highlights the potential of **L13** as a convenient platform for biological applications, as antibodies and peptides conjugated to **L13** will not be affected by the mild conditions.

## 4 Modification of Macrocyclic Sizes

In our latest work, we explored chiral modifications on other macrocyclic chelators with different cavity sizes (Figure 13).^4^ The 9-membered NOTA is the most promising and well-studied macrocyclic ligand after DOTA, thus we designed chiral NOTA **L20** with ethyl substituents for comparison with our benchmark chiral DOTA **L2** (Figure 14). The 10-membered 1,4,7-triazacyclodecane-1,4,7-triacetate acid (DETA) chelator was first developed in 1971 with several follow-up investigations of its structure and basic coordination properties.^124–127^ According to Hancock et al.’s metal ion selectivity theory,^128–130^ DETA’s 6-membered coordination ring might be beneficial for chelating small metal ions like Ga^3+^; nevertheless, studies on DETA complexes are rather limited. Based on DFT calculations, we selected a combination of chiral methyl and ethyl groups for our chiral DETA design,s and both the α- and β-versions of chiral DETA were synthesized, namely, **L21** and **L22**.

**Figure 13:**
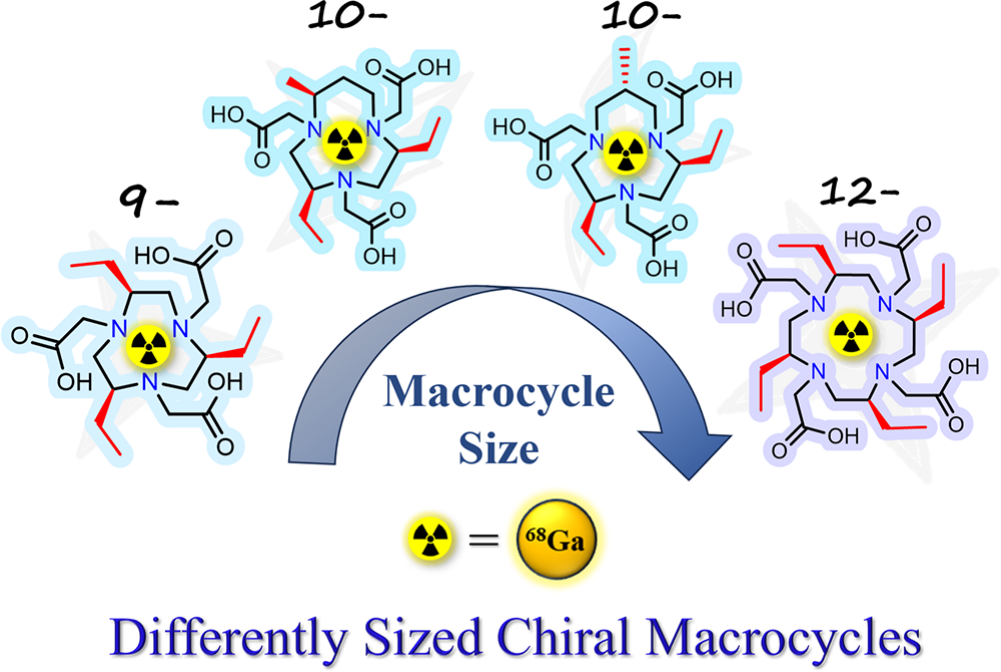
Design of differently sized chiral macrocyclic chelators for ^68^Ga radiolabeling.^4^

**Figure 14:**
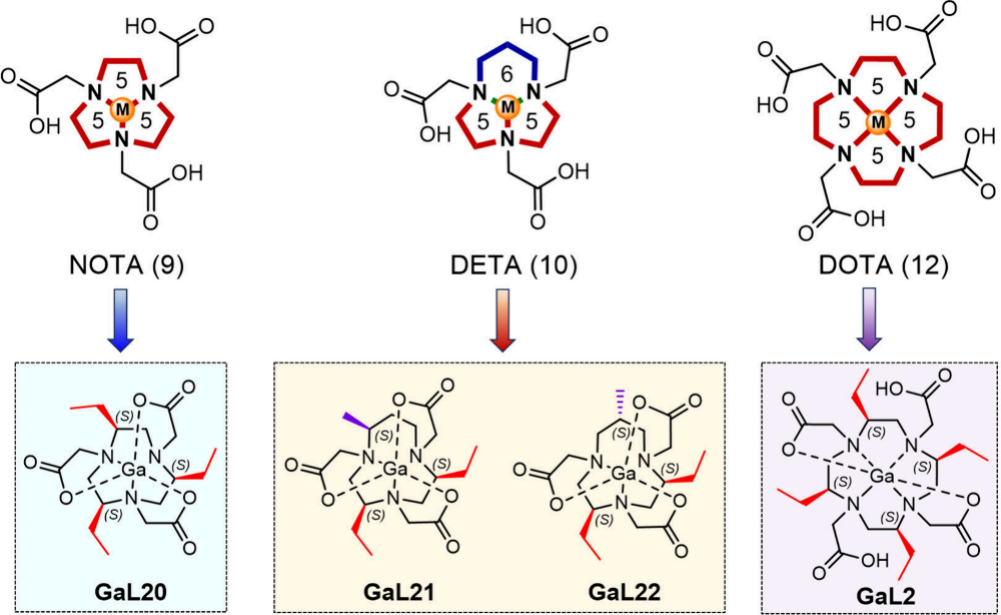
Structures of the Ga^3+^ complexes with the four differently sized chiral macrocyclic ligands and their parent achiral chelators. Adapted from ref 4. Available under a CC BY-NC-ND 4.0 license. Copyright 2025 The Authors.

Unlike chiral DOTAs, chiral NOTA and DETA cannot be synthesized via cyclization of aziridines; new synthetic routes employing chiral amino acid derivatives as building blocks to construct linear intermediates for cyclization were designed. ^68^Ga radiolabeling studies suggested a general RCY (radiochemical yield) trend of **L20** (9) > **L2** (12) > **L21** (α-10) > **L22** (β-10). Chiral NOTA **L20** gave nearly quantitative RCYs under all of the investigated conditions, displaying the best ^68^Ga radiolabeling kinetics among the four chelators. In comparison, the RCYs for the 10- and 12- membered chelators are inferior at room temperature, reaching only >90% conversion at increased temperature. This conveys that fast kinetics is desired for radiolabeling, contrary to properties favoring good complex inertness. To gain deeper insight into the influences of ring sizes and chiral substituents on Ga^3+^ coordination stability, equilibrium studies, DFT calculations, and kinetic stability tests were conducted.

It is well known that the stability of a metal chelate is defined by both kinetic and thermodynamic stability.^131^ We first investigated the basicity of our chiral chelators and the thermodynamic stability of their Ga^3+^ complexes through potentiometric titrations (Table 4). According to the overall protonation constants (∑ log *K*_i_^H^), the basicity of these chiral macrocycles is similar to that of their parent achiral ligands. NMR titration results of **L20** correlate well with its speciation diagram produced from potentiometric titration data, suggesting that the chiral substituents do not affect the protonation sequence of a macrocyclic ligand. In terms of Ga^3+^ stability constants (log *K*_GaL_), a trend of **L20** > **L21** > **L22** > NOTA > **L2** > DOTA was observed, which is further supported by our DFT studies. Subsequent *in vitro* serum stability tests and kinetic studies under acid and alkaline conditions confirmed that the kinetic inertness of **GaL2** and **GaL20–GaL22** is favorable for PET imaging applications.

**Table 4:** Results of Equilibrium Studies and DFT (Density Functional Theory) Calculations for **L2** and **L20–L22** ^4^

	L20^a^	L21^a^	L22^a^	L2^a^	NOTA^b^	DETA^d^	DOTA^e^
log *K*_1_^H^	11.16 ± 0.02	11.42 ± 0.19	10.55 ± 0.16	11.95 ± 0.21	12.00 ± 0.01	14.8 ± 0.18	11.14
log *K*_2_^H^	6.47 ± 0.16	7.57 ± 0.19	6.61 ± 0.01	9.53 ± 0.11	5.65 ± 0.02	6.12 ± 0.02	9.69
log *K*_3_^H^	3.49 ± 0.18	3.77 ± 0.20	4.12 ± 0.11	5.26 ± 0.08	3.19 ± 0.03	3.69 ± 0.02	4.84
log *K*_4_^H^	/	/	/	4.00 ± 0.07	/	/	3.95
∑ log *K*_i_^H^	21.12	22.76	21.28	30.74	20.84	24.61	29.62
log *K*_GaL_	31.20	31.02	30.45	27.26	29.60^c^	/	26.05^f^
ΔG°_B_ of GaL (kcal mol^–1^)	–271.8	–272.0	–270.8	–229.4	–269.5	–268.4	–229.5
Calc. log *K*_GaL_	31.28	31.46	29.79	26.02	/	28.85	/

a I = 0.1 M NMe_4_Cl, 25 °C, where *K*_i_^H^ = [H_i_L]/[H_i‑1_L]­[H^+^]. The stability constant (log *K*_GaL_) values are the averages of two sets of data from two independent measurements.

b I = 0.1 M NMe_4_Cl, 25 °C (from ref 57).

c From ref 85.

d I = 0.1 M KCl, 25 °C (from ref 126).

e I = 0.1 M KCl, 25 °C (from ref 132).

f From ref 133.

Our studies yielded three conclusions: (1) Introducing chiral substituents onto the macrocyclic backbones of NOTA, DETA, and DOTA improved their Ga^3+^ coordination stability. (2) Introducing an *S*-methyl group at the α-position of DETA resulted in more ideal overall ligand basicity and Ga^3+^ chelating affinity than at the β-position. (3) Consistent with Hancock et al.’s theory, the 6-membered ring of chiral DETA can counteract the reduction in Ga^3+^ binding affinity caused by increased macrocyclic cavity size, rendering the stability constants of **L21** and **L22** close to that of **L20** with a smaller macrocycle. As a proof-of-concept study, this work provides insights for the future design of next-generation chiral macrocycles with varied cavity sizes.

## 5 Summary and Outlook

We report a class of novel chiral macrocyclic chelators with different chiral substituents on their backbones as versatile biological probes. We employed three design strategies for developing our chiral ligands: (1) modification of the macrocyclic backbone, (2) modification of the pendant arms, and (3) alteration of the macrocyclic ring size. We started with the less common but most effective approach of macrocyclic backbone modification by introducing chiral groups into the macrocycle framework of DOTA. With enhanced rigidity on the macrocyclic ring, these chiral DOTA ligands prevent the interconversion between geometric isomers, thereby controlling the isomeric ratio or reducing the number of isomers in the resulting lanthanide complexes. The introduced chiral substituents resulted in improved coordination geometry, which endowed our chiral DOTA complexes with significantly higher stability compared to that of the achiral analogues. Proof-of-concept studies revealed their advantageous radiochemical, luminescence, and biocompatibility, highlighting their great potential for different biological applications. We then modified the pendant arm structures of these chiral DOTAs to create various bifunctional chelators (BFCs) for targeted diagnostic and therapeutic applications. Our recent study explored the effect of macrocyclic ring sizes by developing chiral analogues of the 9-membered NOTA and 10-membered DETA chelators for ^68^Ga^3+^ radiolabeling. The observed radiolabeling efficacy and thermodynamic stability trends provide valuable information about the effect of macrocyclic cavity sizes and chiral modifications on the ligand coordination chemistry.

In addition to NOTA, DETA, and DOTA, there are still many differently sized macrocyclic chelators with limited studies, such as the 11-membered 1,4,8-triazacycloundecane-1,4,8-triacetic acid (UNTA)^134,135^ and the 13-membered 1,4,7,10-tetraaza­cyclotridecane-1,4,7,10-tetraacetic acid (TRITA)^132,136–138^ (Figure 15). The lack of reports on these macrocycles arises from the difficult synthesis and purification and has confined the study of their metal coordination chemistry. Although DOTA and NOTA have been regarded as the benchmark or “gold standard” chelator, studies on the less-explored macrocycles should not be neglected. The fundamental design principal of macrocyclic chelators needs to be taken into consideration, such as ligand denticity, rigidity, coordination geometry, and their match with the size of metal ions.^139–141^ Therefore, understanding the metal coordination chemistry in differently sized macrocyclic chelators is of immense importance to gain insight into the structure–property relationships. Our future work will focus on expanding our class of chiral macrocycles by developing other pendant arms as well as exploring other rings such as the 11- and 13-membered macrocycles. We are convinced that these fundamental studies on metal-size selectivity of macrocyclic chelation will pave the way for the future design of next-generation macrocyclic ligands which will serve as a tool to drive applications in metal sequestration and in advanced diagnostic applications.

**Figure 15:**
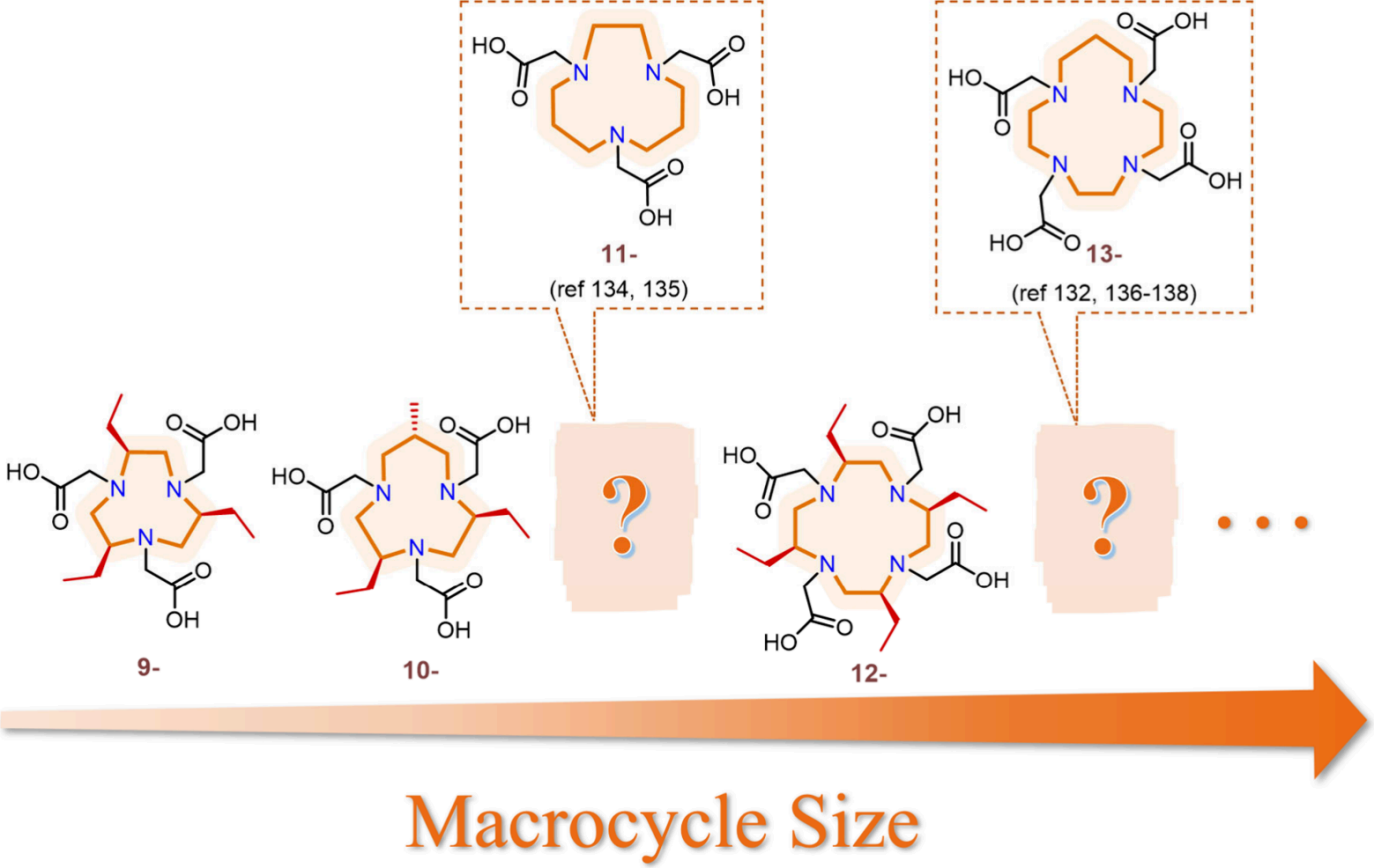
Current research gap of the 11- and 13-membered chiral macrocyclic chelators.^132,134–138^

## References

[1] Dai L., Jones C. M., Chan W. T. K., Pham T. A., Ling X., Gale E. M., Rotile N. J., Tai W. C.-S., Anderson C. J., Caravan P., Law G.-L. (2018). Chiral DOTA chelators as an improved
platform for biomedical imaging and therapy applications. Nat. Commun..

[2] Zhang J., Dai L., Webster A. M., Chan W. T. K., Mackenzie L. E., Pal R., Cobb S. L., Law G. L. (2021). Unusual
magnetic field responsive
circularly polarized luminescence probes with highly emissive chiral
europium (III) complexes. Angew. Chem., Int.
Ed..

[3] Zhang J., Dai L., He L., Bhattarai A., Chan C.-M., Tai W. C.-S., Vardhanabhuti V., Law G.-L. (2023). Design and synthesis
of chiral DOTA-based MRI contrast agents with remarkable relaxivities. Commun. Chem..

[4] Zhu Y., Cheng Z., Zhang J., Feng L., Mishra A., de Rosales R. T. M., Brown R. K., Long N., Law G.-L. (2026). Influences
of Ring Sizes and Chiral Substituents on Macrocyclic Chelators for
Improving ^68^Ga Radiolabeling. JACS
Au.

[5] Kiening M., Lange N. (2022). A recap of heme metabolism towards understanding protoporphyrin IX
selectivity in cancer cells. Int. J. Mol. Sci..

[6] Martins T., Barros A. N., Rosa E., Antunes L. (2023). Enhancing health benefits
through chlorophylls and chlorophyll-rich agro-food: A comprehensive
review. Molecules.

[7] Lindoy L. F., Park K.-M., Lee S. S. (2013). Metals,
macrocycles and molecular
assemblies-macrocyclic complexes in metallo-supramolecular chemistry. Chem. Soc. Rev..

[8] Joshi T., Graham B., Spiccia L. (2015). Macrocyclic
metal complexes for metalloenzyme
mimicry and sensor development. Acc. Chem. Res..

[9] Lindoy, L. F. Chemistry of Macrocyclic Ligand Complexes; Cambridge, 2003.

[10] Gloe, K. Macrocyclic Chemistry; Springer, 2005.

[11] Yu, X. ; Zhang, J. Macrocyclic Polyamines: Synthesis and Applications; John Wiley & Sons, 2018.

[12] Thunus L., Lejeune R. (1999). Overview of transition
metal and lanthanide complexes
as diagnostic tools. Coord. Chem. Rev..

[13] Kostelnik T. I., Orvig C. (2019). Radioactive main group and rare earth metals for imaging and therapy. Chem. Rev..

[14] Boros E., Comba P., Engle J. W., Harriswangler C., Lapi S. E., Lewis J. S., Mastroianni S., Mirica L. M., Platas-Iglesias C., Ramogida C. F. (2025). Chemical Tools to
Characterize the Coordination Chemistry of Radionuclides for Radiopharmaceutical
Applications. Chem. Rev..

[15] Shinoda S. (2013). Dynamic cyclen-metal
complexes for molecular sensing and chirality signaling. Chem. Soc. Rev..

[16] Mishra P., Sethi P., Kumar S., Rathi P., Umar A., Kumar R., Chaudhary S., Alkhanjaf A. A. M., Ibrahim A. A., Baskoutas S. (2024). Synthesis
and biomedical applications
of macrocyclic complexes. J. Mol. Struct..

[17] Pinalli R., Pedrini A., Dalcanale E. (2018). Biochemical
sensing with macrocyclic
receptors. Chem. Soc. Rev..

[18] Liu J., Zhang X., Lu X., Li Q., Xue Z., Zhang Q., Wu G., Xia W. (2025). Review on
extraction
and separation of valuable metal ions from aqueous solution by crown
ether. Chin. J. Chem. Eng..

[19] Stasiuk G. J., Long N. J. (2013). The ubiquitous DOTA
and its derivatives: the impact
of 1, 4, 7, 10-tetraazacyclododecane-1, 4, 7, 10-tetraacetic acid
on biomedical imaging. Chem. Commun..

[20] Price E. W., Orvig C. (2014). Matching chelators
to radiometals for radiopharmaceuticals. Chem.
Soc. Rev..

[21] Oudkerk M., Sijens P. E., Van Beek E. J., Kuijpers T. J. (1995). Safety and efficacy
of Dotarem (Gd-DOTA) versus magnevist (Gd-DTPA) in magnetic resonance
imaging of the central nervous system. Invest.
Radiol..

[22] Wadas T. J., Wong E. H., Weisman G. R., Anderson C. J. (2010). Coordinating radiometals
of copper, gallium, indium, yttrium, and zirconium for PET and SPECT
imaging of disease. Chem. Rev..

[23] Ariztia J., Solmont K., Moise N. P., Specklin S., Heck M. P., Lamande-Langle S., Kuhnast B. (2022). PET/fluorescence imaging: An overview
of the chemical strategies to build dual imaging tools. Bioconjugate Chem..

[24] Du Y., Semghouli A., Wang Q., Mei H., Kiss L., Baecker D., Soloshonok V. A., Han J. (2025). FDA-approved drugs
featuring macrocycles or medium-sized rings. Arch. Pharm..

[25] Robert P., Lehericy S., Grand S., Violas X., Fretellier N., Idée J.-M., Ballet S., Corot C. (2015). T1-weighted hypersignal
in the deep cerebellar nuclei after repeated administrations of gadolinium-based
contrast agents in healthy rats: difference between linear and macrocyclic
agents. Invest. Radiol..

[26] Robert P., Violas X., Grand S., Lehericy S., Idée J.-M., Ballet S., Corot C. (2016). Linear gadolinium-based
contrast
agents are associated with brain gadolinium retention in healthy rats. Invest. Radiol..

[27] Bussi S., Coppo A., Botteron C., Fraimbault V., Fanizzi A., De Laurentiis E., Colombo Serra S., Kirchin M. A., Tedoldi F., Maisano F. (2018). Differences
in gadolinium
retention after repeated injections of macrocyclic MR contrast agents
to rats. J. Magn. Reson. Imaging.

[28] Tei L., Baranyai Z., Gaino L., Forgács A., Vágner A., Botta M. (2015). Thermodynamic stability,
kinetic
inertness and relaxometric properties of monoamide derivatives of
lanthanide (III) DOTA complexes. Dalton Trans..

[29] Stasiuk G.
J., Long N. J. (2013). The ubiquitous
DOTA and its derivatives: the impact
of 1,4,7,10-tetraazacyclododecane-1,4,7,10-tetraacetic acid on biomedical
imaging. Chem. Commun..

[30] Caravan P., Ellison J. J., McMurry T. J., Lauffer R. B. (1999). Gadolinium (III)
chelates as MRI contrast agents: structure, dynamics, and applications. Chem. Rev..

[31] Clough T. J., Jiang L., Wong K.-L., Long N. J. (2019). Ligand design strategies
to increase stability of gadolinium-based magnetic resonance imaging
contrast agents. Nat. Commun..

[32] Baranyai Z., Tircsó G., Rösch F. (2020). The use of the macrocyclic chelator
DOTA in radiochemical separations. Eur. J. Inorg.
Chem..

[33] Li H., Meade T. J. (2019). Molecular magnetic resonance imaging with Gd (III)-based
contrast agents: challenges and key advances. J. Am. Chem. Soc..

[34] Li M., Wang S., Kong Q., Cheng X., Yan H., Xing Y., Lan X., Jiang D. (2023). Advances in macrocyclic
chelators for positron emission tomography imaging. View.

[35] Stefania R., Palagi L., Di Gregorio E., Ferrauto G., Dinatale V., Aime S., Gianolio E. (2024). Seeking for Innovation with magnetic
resonance imaging paramagnetic contrast agents: relaxation enhancement
via weak and dynamic electrostatic interactions with positively charged
groups on endogenous macromolecules. J. Am.
Chem. Soc..

[36] Qi S., Hoppmann S., Xu Y., Cheng Z. (2019). PET imaging of HER2-positive
tumors with Cu-64-labeled affibody molecules. Mol. Imaging Biol..

[37] Woods M., Kovacs Z., Zhang S., Sherry A. D. (2003). Towards
the Rational
Design of Magnetic Resonance Imaging Contrast Agents: Isolation of
the Two Coordination Isomers of Lanthanide DOTA-Type Complexes. Angew. Chem., Int. Ed..

[38] Miao Q., Dekkers R., Gupta K. B. S. S., Overhand M., Dasgupta R., Ubbink M. (2023). Rigidified and Hydrophilic
DOTA-like Lanthanoid Ligands:
Design, Synthesis, and Dynamic Properties. Inorg.
Chem..

[39] Caravan P. (2006). Strategies
for increasing the sensitivity of gadolinium based MRI contrast agents. Chem. Soc. Rev..

[40] Hao D., Ai T., Goerner F., Hu X., Runge V. M., Tweedle M. (2012). MRI contrast
agents: basic chemistry and safety. J. Magn.
Reson. Imaging.

[41] Mishiro K., Imai S., Ematsu Y., Hirose K., Fuchigami T., Munekane M., Kinuya S., Ogawa K. (2022). RGD peptide-conjugated
dodecaborate with the Ga-DOTA complex: a preliminary study for the
development of theranostic agents for boron neutron capture therapy
and its companion diagnostics. J. Med. Chem..

[42] Park J.-A., Lee J.-J., Jung J.-C., Yu D.-Y., Oh C., Ha S., Kim T.-J., Chang Y. (2008). Gd-DOTA Conjugate of RGD as a Potential
Tumor-Targeting MRI Contrast Agent. ChemBioChem..

[43] Xu W., Lu Y., Xu J., Li H., Lan R., Gao R., Ding Y., Ye X., Shu K., Ye F. (2023). Rational Design of Gd-DOTA-Type
Contrast Agents for Hepatobiliary
Magnetic Resonance Imaging. J. Med. Chem..

[44] McQuade P., Miao Y., Yoo J., Quinn T. P., Welch M. J., Lewis J. S. (2005). Imaging of Melanoma Using ^64^Cu- and ^86^Y-DOTA-ReCCMSH­(Arg^11^), a Cyclized Peptide Analogue
of α-MSH. J. Med. Chem..

[45] Jin W., Zhao R., Wang R., Choi S. R., Ploessl K., Alexoff D., Wu Z., Zhu L., Kung H. F. (2024). Theranostic
Agent Targeting Bone Metastasis: A Novel [^68^Ga]­Ga/[^177^Lu]­Lu-DOTA-HBED-bisphosphonate. J.
Med. Chem..

[46] Guo X., Xu C., Zhu X., Wang X., Hao Y., Wang X., Yao Y., Fang J., Wang K. (2025). In Silico
Design and Evaluation of
Novel Peptide-Based PET Probe for Noninvasive Imaging of HER2 Expression
in Breast Cancer. J. Med. Chem..

[47] Kolenc-Peitl P., Mansi R., Tamma M., Gmeiner-Stopar T., Sollner-Dolenc M., Waser B., Baum R. P., Reubi J. C., Maecke H. R. (2011). Highly Improved Metabolic Stability and Pharmacokinetics
of Indium-111-DOTA-Gastrin Conjugates for Targeting of the Gastrin
Receptor. J. Med. Chem..

[48] Notni J., Hermann P., Dregely I., Wester H.-J. (2013). Convenient Synthesis
of ^68^Ga-Labeled Gadolinium­(III) Complexes: Towards Bimodal
Responsive Probes for Functional Imaging with PET/MRI. Chem.—Eur. J..

[49] Park J.-A., Kim J. Y., Lee Y. J., Lee W., Lim S. M., Kim T.-J., Yoo J., Chang Y., Kim K. M. (2013). Gadolinium
Complex of ^125^I/^127^I-RGD-DOTA Conjugate as a
Tumor-Targeting SPECT/MR Bimodal Imaging Probe. ACS Med. Chem. Lett..

[50] Vanasschen C., Bouslimani N., Thonon D., Desreux J. F. (2011). Gadolinium DOTA
Chelates Featuring Alkyne Groups Directly Grafted on the Tetraaza
Macrocyclic Ring: Synthesis, Relaxation Properties, “Click”
Reaction, and High-Relaxivity Micelles. Inorg.
Chem..

[51] Maimouni I., Henoumont C., De Goltstein M.-C., Mayer J.-F., Dehimi A., Boubeguira Y., Kattenbeck C., Maas T. J., Decout N., Strzeminska I. (2025). Gadopiclenol: A q= 2 gadolinium-based MRI contrast
agent combining high stability and efficacy. Invest. Radiol..

[52] Robic C., Port M., Rousseaux O., Louguet S., Fretellier N., Catoen S., Factor C., Le Greneur S., Medina C., Bourrinet P. (2019). Physicochemical
and pharmacokinetic
profiles of gadopiclenol: a new macrocyclic gadolinium chelate with
high T1 relaxivity. Invest. Radiol..

[53] Hancock R. D., Martell A. E. (1989). Ligand design for
selective complexation of metal ions
in aqueous solution. Chem. Rev..

[54] Martell A. E., Motekaitis R. J., Clarke E. T., Delgado R., Sun Y., Ma R. (1996). Stability constants of metal complexes of macrocyclic ligands with
pendant donor groups. Supramol. Chem..

[55] Sun Y., Anderson C. J., Pajeau T. S., Reichert D. E., Hancock R. D., Motekaitis R. J., Martell A. E., Welch M. J. (1996). Indium (III) and
gallium (III) complexes of bis (aminoethanethiol) ligands with different
denticities: stabilities, molecular modeling, and in vivo behavior. J. Med. Chem..

[56] Boros E., Ferreira C. L., Cawthray J. F., Price E. W., Patrick B. O., Wester D. W., Adam M. J., Orvig C. (2010). Acyclic chelate with
ideal properties for 68Ga PET imaging agent elaboration. J. Am. Chem. Soc..

[57] Clarke E. T., Martell A. E. (1991). Stabilities of the
Fe (III), Ga (III) and In (III)
chelates of N, N′, N ″-triazacyclononanetriacetic acid. Inorg. Chim. Acta.

[58] Zhang Y., Hong H., Engle J. W., Bean J., Yang Y., Leigh B. R., Barnhart T. E., Cai W. (2011). Positron emission tomography
imaging of CD105 expression with a ^64^Cu-labeled monoclonal
antibody: NOTA is superior to DOTA. PLoS One.

[59] Bevilacqua A., Gelb R. I., Hebard W. B., Zompa L. J. (1987). Equilibrium and
thermodynamic study of the aqueous complexation of 1, 4, 7-triazacyclononane-N,
N’, N’’-triacetic acid with protons, alkaline-earth-metal
cations, and copper (II). Inorg. Chem..

[60] Li D., Zhao X., Zhang L., Li F., Ji N., Gao Z., Wang J., Kang P., Liu Z., Shi J. (2014). ^68^Ga-PRGD2 PET/CT in the evaluation of glioma: a prospective study. Mol. Pharmaceutics.

[61] Zheng Y., Wang H., Tan H., Cui X., Yao S., Zang J., Zhang L., Zhu Z. (2019). Evaluation
of lung
cancer and neuroendocrine neoplasm in a single scan by targeting both
somatostatin receptor and integrin αvβ3. Clin. Nucl. Med..

[62] Marlin A., Koller A., Madarasi E., Cordier M., Esteban-Gómez D., Platas-Iglesias C., Tircsó G., Boros E., Patinec V., Tripier R. (2023). H_3_ nota Derivatives Possessing Picolyl and
Picolinate Pendants for Ga^3+^ Coordination and ^67^Ga^3+^ Radiolabeling. Inorg. Chem..

[63] Mewis R. E., Archibald S. J. (2010). Biomedical applications of macrocyclic ligand complexes. Coord. Chem. Rev..

[64] Anderson, C. J. ; Dehdashti, F. ; Cutler, P. D. ; Schwarz, S. W. ; Laforest, R. ; Bass, L. A. ; Lewis, J. S. ; McCarthy, D. W. ^64^Cu-TETA-octreotide as a PET imaging agent for patients with neuroendocrine tumors. J. Nucl. Med. 2001, 42(2), 213–221. 11216519

[65] Clarke E. T., Martell A. E. (1991). Stabilities of the alkaline earth
and divalent transition
metal complexes of the tetraazamacrocyclic tetraacetic acid ligands. Inorg. Chim. Acta.

[66] Reissig F., Bauer D., Zarschler K., Novy Z., Bendova K., Ludik M.-C., Kopka K., Pietzsch H.-J., Petrik M., Mamat C. (2021). Towards targeted alpha
therapy with actinium-225: chelators for mild
condition radiolabeling and targeting PSMA—a proof of concept
study. Cancers (Basel).

[67] Nelson B. J., Andersson J. D., Wuest F. (2022). Radiolanthanum: Promising theranostic
radionuclides for PET, alpha, and Auger-Meitner therapy. Nucl. Med. Biol..

[68] Ahenkorah S., Cassells I., Deroose C. M., Cardinaels T., Burgoyne A. R., Bormans G., Ooms M., Cleeren F. (2021). Bismuth-213
for targeted radionuclide therapy: from atom to bedside. Pharmaceutics.

[69] Reissig F., Bauer D., Ullrich M., Kreller M., Pietzsch J., Mamat C., Kopka K., Pietzsch H.-J., Walther M. (2020). Recent insights
in barium-131 as a diagnostic match for radium-223: cyclotron production,
separation, radiolabeling, and imaging. Pharmaceuticals.

[70] Abou D. S., Thiele N. A., Gutsche N. T., Villmer A., Zhang H., Woods J. J., Baidoo K. E., Escorcia F. E., Wilson J. J., Thorek D. L. (2021). Towards the stable
chelation of radium for biomedical
applications with an 18-membered macrocyclic ligand. Chem. Sci..

[71] Blei M. K., Waurick L., Reissig F., Kopka K., Stumpf T., Drobot B. r., Kretzschmar J., Mamat C. (2023). Equilibrium thermodynamics
of macropa complexes with selected metal isotopes of radiopharmaceutical
interest. Inorg. Chem..

[72] Kovács A. (2020). Theoretical
study of actinide complexes with macropa. ACS
Omega.

[73] Woods M., Aime S., Botta M., Howard J. A., Moloney J. M., Navet M., Parker D., Port M., Rousseaux O. (2000). Correlation
of water exchange rate with isomeric composition in diastereoisomeric
gadolinium complexes of tetra (carboxyethyl) dota and related macrocyclic
ligands. J. Am. Chem. Soc..

[74] Aime S., Botta M., Fasano M., Marques M. P. M., Geraldes C. F. G. C., Pubanz D., Merbach A. E. (1997). Conformational
and Coordination Equilibria
on DOTA Complexes of Lanthanide Metal Ions in Aqueous Solution Studied
by ^1^H-NMR Spectroscopy. Inorg. Chem..

[75] Meyer M., Dahaoui-Gindrey V., Lecomte C., Guilard R. (1998). Conformations and coordination
schemes of carboxylate and carbamoyl derivatives of the tetraazamacrocycles
cyclen and cyclam, and the relation to their protonation states. Coord. Chem. Rev..

[76] Jacques V., Desreux J. F. (1994). Quantitative Two-Dimensional EXSY
Spectroscopy and
Dynamic Behavior of a Paramagnetic Lanthanide Macrocyclic Chelate:
YbDOTA­(DOTA = 1,4,7,10-Tetraazacyclododecane-N,N’,N’’,N’’’-tetraacetic
Acid). Inorg. Chem..

[77] Desreux J. F. (1980). Nuclear
magnetic resonance spectroscopy of lanthanide complexes with a tetraacetic
tetraaza macrocycle. Unusual conformation properties. Inorg. Chem..

[78] Aime S., Barge A., Botta M., Parker D., De Sousa A. S. (1997). Prototropic
vs whole water exchange contributions to the solvent relaxation enhancement
in the aqueous solution of a cationic Gd^3+^ macrocyclic
complex. J. Am. Chem. Soc..

[79] Aime S., Barge A., Bruce J. I., Botta M., Howard J. A., Moloney J. M., Parker D., De Sousa A. S., Woods M. (1999). NMR, relaxometric,
and structural studies of the hydration and exchange dynamics of cationic
lanthanide complexes of macrocyclic tetraamide ligands. J. Am. Chem. Soc..

[80] Dunand F. A., Aime S., Merbach A. E. (2000). First ^17^O NMR Observation
of Coordinated Water on Both Isomers of [Eu­(DOTAM)­(H_2_O)]^3+^: A Direct Access to Water Exchange and its Role in the Isomerization^1^. J. Am. Chem. Soc..

[81] Vlasie M. D., Comuzzi C., van den
Nieuwendijk A.
M., Prudêncio M., Overhand M., Ubbink M. (2007). Long-range-distance NMR effects in
a protein labeled with a lanthanide-DOTA chelate. Chem.—Eur. J..

[82] Woods M., Kovacs Z., Zhang S., Sherry A. D. (2003). Towards the rational
design of magnetic resonance imaging contrast agents: isolation of
the two coordination isomers of lanthanide DOTA-type complexes. Angew. Chem., Int. Ed..

[83] Geraldes C. F., Marques M. P. M., Sherry A. D. (1998). NMR conformational
study of diamagnetic
complexes of some triazatriacetate macrocycles. Inorg. Chim. Acta.

[84] Geraldes C., Alpoim M., Marques M., Sherry A. D., Singh M. (1985). Nuclear magnetic
resonance and potentiometric studies of the protonation scheme of
a triaza triacetic macrocycle and its complexes with lanthanum and
lutetium. Inorg. Chem..

[85] Simecek J., Schulz M., Notni J., Plutnar J., Kubicek V., Havlickova J., Hermann P. (2012). Complexation of metal ions with TRAP
(1,4,7-triazacyclononane phosphinic acid) ligands and 1,4,7-triazacyclononane-1,4,7-triacetic
acid: phosphinate-containing ligands as unique chelators for trivalent
gallium. Inorg. Chem..

[86] Ranganathan R. S., Pillai R. K., Raju N., Fan H., Nguyen H., Tweedle M. F., Desreux J. F., Jacques V. (2002). Polymethylated
DOTA
ligands. 1. synthesis of rigidified ligands and studies on the effects
of alkyl substitution on acid- base properties and conformational
mobility. Inorg. Chem..

[87] Ranganathan R. S., Raju N., Fan H., Zhang X., Tweedle M. F., Desreux J. F., Jacques V. (2002). Polymethylated
DOTA Ligands. 2. Synthesis
of Rigidified Lanthanide Chelates and Studies on the Effect of Alkyl
Substitution on Conformational Mobility and Relaxivity. Inorg. Chem..

[88] Howard J. K., Kenwright A., Moloney J. (1998). Structure and dynamics of all of
the stereoisomers of europium complexes of tetra (carboxyethyl) derivatives
of dota: ring inversion is decoupled from cooperative arm rotation
in the *RRRR* and *RRRS* isomers. Chem. Commun..

[89] Anelli P. L., Beltrami A., Franzini M., Paoli P., Rossi P., Uggeri F., Virtuani M. (2001). Gd (III) complexes of poly (hydroxymethyl)
substituted derivatives of 1, 4, 7, 10-tetraazacyclododecane-1, 4,
7, 10-tetraacetic acid. Inorg. Chim. Acta.

[90] Kamioka S., Takahashi T., Kawauchi S., Adachi H., Mori Y., Fujii K., Uekusa H., Doi T. (2009). Chiral tetraazamacrocycles
having four pendant-arms. Org. Lett..

[91] Dai L., Zhang J., Chen Y., Mackenzie L. E., Pal R., Law G.-L. (2019). Synthesis of water-soluble
chiral DOTA lanthanide complexes
with predominantly twisted square antiprism isomers and circularly
polarized luminescence. Inorg. Chem..

[92] Tircso G., Webber B. C., Kucera B. E., Young V. G., Woods M. (2011). Analysis of
the conformational behavior and stability of the SAP and TSAP isomers
of lanthanide (III) NB-DOTA-type chelates. Inorg.
Chem..

[93] Aime S., Barge A., Botta M., De Sousa A. S., Parker D. (1998). Direct NMR
spectroscopic observation of a lanthanide-coordinated water molecule
whose exchange rate is dependent on the conformation of the complexes. Angew. Chem., Int. Ed..

[94] Carr R., Evans N. H., Parker D. (2012). Lanthanide
complexes as chiral probes
exploiting circularly polarized luminescence. Chem. Soc. Rev..

[95] Kumar J., Nakashima T., Kawai T. (2015). Circularly polarized luminescence
in chiral molecules and supramolecular assemblies. J. Phys. Chem. Lett..

[96] Zhao X., Zang S.-Q., Chen X. (2020). Stereospecific
interactions between
chiral inorganic nanomaterials and biological systems. Chem. Soc. Rev..

[97] Shuvaev S., Fox M. A., Parker D. (2018). Monitoring
of the ADP/ATP ratio by
induced circularly polarised europium luminescence. Angew. Chem..

[98] Zinna F., Di Bari L. (2015). Lanthanide circularly
polarized luminescence: bases
and applications. Chirality.

[99] Leonzio M., Melchior A., Faura G., Tolazzi M., Zinna F., Di Bari L., Piccinelli F. (2017). Strongly circularly
polarized emission
from water-soluble Eu (III)-and Tb (III)-based complexes: a structural
and spectroscopic study. Inorg. Chem..

[100] Zhang Z., Kolodziej A. F., Greenfield M. T., Caravan P. (2011). Heteroditopic binding
of MR contrast agents for increased
relaxivity. Angew. Chem., Int. Ed. Engl..

[101] Tei L., Barge A., Galli M., Pinalli R., Lattuada L., Gianolio E., Aime S. (2015). Polyhydroxylated
GdDTPA-derivatives
as high relaxivity magnetic resonance imaging contrast agents. RSC Adv..

[102] Caravan P., Esteban-Gómez D., Rodríguez-Rodríguez A., Platas-Iglesias C. (2019). Water exchange
in lanthanide complexes for MRI applications.
Lessons learned over the last 25 years. Dalton
Trans..

[103] Dai L., Zhang J., Wong C. T., Chan W. T. K., Ling X., Anderson C. J., Law G.-L. (2021). Design of functional chiral cyclen-based
radiometal chelators for theranostics. Inorg.
Chem..

[104] Dickins R. S., Howard J. A., Maupin C. L., Moloney J. M., Parker D., Riehl J. P., Siligardi G., Williams J. G. (1999). Synthesis, time-resolved luminescence, NMR spectroscopy,
circular dichroism and circularly polarised luminescence studies of
enantiopure macrocyclic lanthanide tetraamide complexes. Chem.—Eur. J..

[105] Regueiro-Figueroa M., Bensenane B., Ruscsák E., Esteban-Gómez D., Charbonnière L.
J., Tircsó G., Tóth I., Blas A. d., Rodríguez-Blas T., Platas-Iglesias C. (2011). Lanthanide dota-like Complexes Containing a Picolinate
Pendant: Structural Entry for the Design of LnIII-Based Luminescent
Probes. Inorg. Chem..

[106] Martins A. F., Eliseeva S. V., Carvalho H. F., Teixeira J. M., Paula C. T., Hermann P., Platas-Iglesias C., Petoud S., Tóth É., Geraldes C. F. (2014). A Bis­(pyridine *N*-oxide) Analogue of DOTA: Relaxometric Properties of the
Gd^III^ Complex and Efficient Sensitization of Visible and
NIR-Emitting Lanthanide­(III) Cations Including Pr^III^ and
Ho^III^. Chem.—Eur. J..

[107] Hennrich U., Benešová M. (2020). [^68^Ga] Ga-DOTA-TOC:
the first FDA-approved ^68^Ga-radiopharmaceutical for PET
imaging. Pharmaceuticals.

[108] Hu K., Ma X., Xie L., Zhang Y., Hanyu M., Obata H., Zhang L., Nagatsu K., Suzuki H., Shi R. (2022). Development of a stable
peptide-based PET tracer for detecting CD133-expressing
cancer cells. ACS Omega.

[109] Zhao R., Ploessl K., Zha Z., Choi S., Alexoff D., Zhu L., Kung H. F. (2020). Synthesis
and Evaluation
of ^68^Ga- and ^177^Lu-Labeled (*R*)- vs (*S*)-DOTAGA Prostate-Specific Membrane Antigen-Targeting
Derivatives. Mol. Pharmaceutics.

[110] Tu C., Louie A. Y. (2007). Photochromically-controlled, reversibly-activated MRI
and optical contrast agent. Chem. Commun..

[111] Kruttwig K., Yankelevich D. R., Brueggemann C., Tu C., L’Etoile N., Knoesen A., Louie A. Y. (2012). Reversible
low-light induced photoswitching of crowned spiropyran-DO3A complexed
with gadolinium (III) ions. Molecules.

[112] Kitamura M., Suzuki T., Abe R., Ueno T., Aoki S. (2011). ^11^B NMR Sensing of d-Block Metal Ions in Vitro and in
Cells Based on the Carbon-Boron Bond Cleavage of Phenylboronic Acid-Pendant
Cyclen (Cyclen = 1,4,7,10-Tetraazacyclododecane). Inorg. Chem..

[113] Kölmel D. K., Fürniss D., Susanto S., Lauer A., Grabher C., Bräse S., Schepers U. (2012). Cell penetrating peptoids
(CPPos): Synthesis of a small combinatorial library by using IRORI
MiniKans. Pharmaceuticals.

[114] Bellis S. L. (2011). Advantages of RGD peptides for directing
cell association
with biomaterials. Biomaterials.

[115] Alipour M., Baneshi M., Hosseinkhani S., Mahmoudi R., Jabari Arabzadeh A., Akrami M., Mehrzad J., Bardania H. (2020). Recent progress in
biomedical applications of RGD-based
ligand: from precise cancer theranostics to biomaterial engineering:
a systematic review. J. Biomed. Mater. Res.,
Part A.

[116] Ye Y., Bloch S., Xu B., Achilefu S. (2006). Design, synthesis,
and evaluation of near infrared fluorescent multimeric RGD peptides
for targeting tumors. J. Med. Chem..

[117] Deng F., Liu Y. (2012). Study of the interaction
between
tosufloxacin tosylate and bovine serum albumin by multi-spectroscopic
methods. J. Lumin..

[118] Beeby A., Clarkson I. M., Dickins R. S., Faulkner S., Parker D., Royle L., De Sousa A. S., Williams J. G., Woods M. (1999). Non-radiative deactivation of the
excited states of europium, terbium
and ytterbium complexes by proximate energy-matched OH, NH and CH
oscillators: an improved luminescence method for establishing solution
hydration states. J. Chem. Soc., Perkin Trans.
2.

[119] Supkowski R. M., Horrocks W. D. (2002). On the determination
of the number of water molecules, q, coordinated to europium (III)
ions in solution from luminescence decay lifetimes. Inorg. Chim. Acta.

[120] Tanner P. A. (2013). Some misconceptions concerning the electronic spectra
of tri-positive europium and cerium. Chem. Soc.
Rev..

[121] Heffern M. C., Matosziuk L. M., Meade T. J. (2014). Lanthanide probes
for bioresponsive imaging. Chem. Rev..

[122] Ishii K., Hattori S., Kitagawa Y. (2020). Recent advances
in
studies on the magneto-chiral dichroism of organic compounds. Photochem. Photobiol. Sci..

[123] Kitagawa Y., Tsurui M., Hasegawa Y. (2020). Steric and
electronic
control of chiral Eu (III) complexes for effective circularly polarized
luminescence. ACS Omega.

[124] Koyama H., Yoshino T. (1972). Syntheses of some medium
sized cyclic
triamines and their cobalt (III) complexes. Bull. Chem. Soc. Jpn..

[125] Notni J., Pohle K., Peters J. A., Görls H., Platas-Iglesias C. (2009). Structural study of Ga (III), In
(III), and Fe (III)
complexes of triaza-macrocycle based ligands with N_3_S_3_ donor set. Inorg. Chem..

[126] Brucher E., Cortes S., Chavez F., Sherry A. (1991). Synthesis,
equilibrium, and kinetic properties of the gadolinium (III) complexes
of three triazacyclodecanetriacetate ligands. Inorg. Chem..

[127] Majkowska-Pilip A., Bilewicz A. (2011). Macrocyclic complexes of scandium
radionuclides as precursors for diagnostic and therapeutic radiopharmaceuticals. J. Inorg. Biochem..

[128] Hancock R. D. (1986). Macrocycles and their selectivity
for metal ions on
the basis of size. Pure Appl. Chem..

[129] Hancock R. D. (1990). Molecular
mechanics calculations and metal ion recognition. Acc. Chem. Res..

[130] Hancock R. D. (1992). Chelate ring size and metal ion selection. The basis
of selectivity for metal ions in open-chain ligands and macrocycles. J. Chem. Educ..

[131] Muthaiah S., Bhatia A., Kannan M. (2020). Stability of metal
complexes. BOOK: Stability and Applications
of Coordination Compounds.

[132] Clarke E. T., Martell A. E. (1991). Stabilities of trivalent metal ion
complexes of the tetraacetate derivatives of 12-, 13-and 14-membered
tetraazamacrocycles. Inorg. Chim. Acta.

[133] Kubicek V., Havlickova J., Kotek J., Tircso G., Hermann P., Toth E., Lukes I. (2010). Gallium­(III) complexes
of DOTA and DOTA-monoamide: kinetic and thermodynamic studies. Inorg. Chem..

[134] Geraldes C. F. G. C., Sherry A. D., Marques M. P. M., Alpoim M. C., Cortes S. (1991). Protonation scheme for some triaza
macrocycles studied
by potentiometry and NMR spectroscopy. J. Chem.
Soc., Perkin Trans. 2.

[135] Camorali S., Nucera A., Saccone M., Carniato F., Botta M., Blasi F., Baranyai Z., Tei L. (2025). The timeless
relevance of size-match selectivity in macrocyclic Fe­(iii) complexes. Inorg. Chem. Front..

[136] Stetter H., Frank W., Mertens R. (1981). Darstellung
und komplexbildung
von polyazacycloalkan-N-essigsäuren. Tetrahedron.

[137] Delgado R., Da Silva J. F. (1982). Metal complexes of cyclic tetra-azatetra-acetic
acids. Talanta.

[138] Pandya D. N., Henry K. E., Day C. S., Graves S. A., Nagle V. L., Dilling T. R., Sinha A., Ehrmann B. M., Bhatt N. B., Menda Y. (2020). Polyazamacrocycle ligands
facilitate ^89^Zr radiochemistry and yield ^89^Zr
complexes with
remarkable stability. Inorg. Chem..

[139] Geduhn J., Walenzyk T., Konig B. (2007). Transition metal complexes
of some azamacrocycles and their use in molecular recognition. Curr. Org. Synth..

[140] Price T. W., Greenman J., Stasiuk G. J. (2016). Current
advances
in ligand design for inorganic positron emission tomography tracers ^68^Ga, ^64^Cu, ^89^Zr and ^44^Sc. Dalton Trans..

[141] Sun Z., Tang H., Wang L., Cao D. (2025). Advances in chiral
macrocycles: molecular design and applications. Chem.—Eur. J..

